# An experimental robotic cell for the disassembly of electric vehicle battery modules

**DOI:** 10.1007/s13243-025-00156-9

**Published:** 2025-10-03

**Authors:** Chaozhi Liang, Anselmo Parnada, Adeyemisi Gbadebo, Faraj Altumi, Mo Qu, Linxiao Li, Ashwin Danny Gomes, Xiaoguo Lin, Duc Truong Pham, Yongjing Wang

**Affiliations:** 1 Department of Mechanical Engineering, School of Engineering, University of Birmingham, Edgbaston, Birmingham, B15 2TT UK; 2 Gomes Technologies, Birmingham, UK

**Keywords:** Robotic disassembly, Battery module, Prototype design, Remanufacturing

## Abstract

The increasing shift towards electrification in the automotive industry has highlighted the critical need for a large-scale and efficient processing of end-of-life (EoL) lithium-ion batteries (LiBs) from electric vehicles (EVs). Linear EoL strategies, such as landfilling, pose significant environmental hazards, while recycling, repurposing, and remanufacturing offer more sustainable alternatives; however, these alternatives require scalable, safe, and cost-efficient disassembly processes. To support circular EoL practices, a prototype of a robotic cell was proposed, presenting a novel automated disassembly process for EV-LiB modules, addressing challenges in the areas of safety and cost-effectiveness. Safety was addressed by encapsulating the work area with protective windows and implementing a safety system that suspends operation if safety conditions are not met. Cost-effectiveness was achieved by implementing a cell layout and robot program that can easily be reconfigured to be compatible with a range of Battery module designs available in the market, and utilising low-cost tools. This paper also presents a methodology for assessing the economic impact of the robotic cell. Initial assessments conducted on a mock-up Samsung model 12S1P Battery module demonstrate that the robotic cell can match the productivity of 5.50 workers within a workweek, saving over £15,000 per month in operational expenses, leading to a potential payback period of 1.33 years. This research marks a step towards realising scalable and financially feasible robotic disassembly systems for EoL EV-LiBs, promoting a circular economy in the automotive sector.

## Introduction

A significant challenge facing the automotive industry is the large-scale processing of end-of-life (EoL) lithium-ion batteries (LiBs), driven by the retirement of LiBs from first-generation electric vehicles (EVs) and the accelerating shift toward electrification [1]. Landfilling, the most common end-of-life (EoL) strategy, while relatively easy to implement at scale, presents serious drawbacks, including environmental pollution, fire-related hazards [2], and the inability to support a sustainable battery supply chain.

**Fig. 1:**
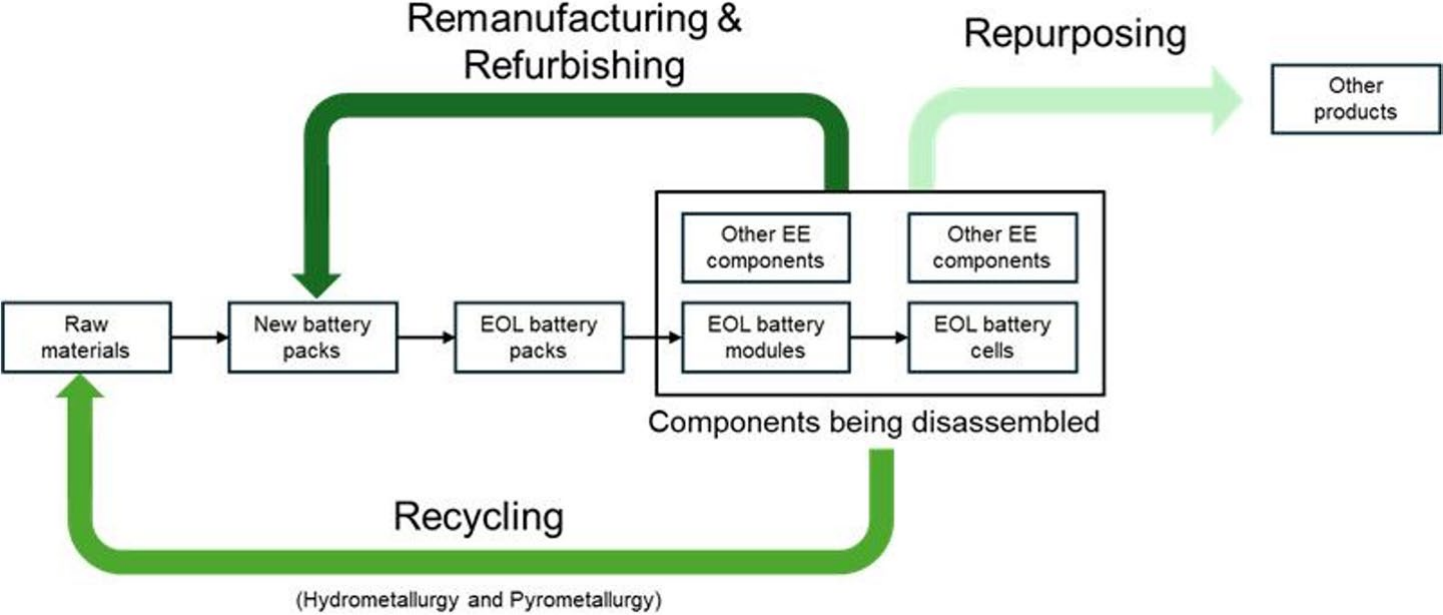
A visual representation of the hierarchical EoL strategy to process EoL EV-LiBs [3]

A hierarchical circular EoL processing strategy has thus been proposed for EV-LiBs, which includes remanufacturing, refurbishing, repurposing, and recycling [3] (Fig. 1). This strategy facilitates the recovery of finite materials, minimises environmental damage, and maximises opportunities for significant cost savings and revenue growth from recirculating materials and product components in the supply chain [4]. This hierarchical strategy proposes multiple pathways, listed below, depending on the condition of the EoL product or the financial conditions on the supplier’s or consumer’s end.

- **Remanufacturing** is an industrial process that restores a used product to a like-new condition, meeting or exceeding the original manufacturer’s specifications in terms of performance, quality, and warranty. It involves disassembling the product, inspecting its components, replacing or restoring any necessary components, reassembling the product, and then testing it to ensure it performs like new. This is typically done if the restoration of a product’s core(s) - a high-value subsystem or component that serves a significant role in the product’s functionality - is more cost-effective than manufacturing a new one, allowing for a “new” product to be produced more cheaply. For EV-LiBs, the cores are the battery modules or cells, and the remanufactured product is a pack with a like-new battery condition.
- **Refurbishing** is similar to remanufacturing, but the resulting product does not have the same warranty or performance as a new one. The restoration of a refurbished product is typically limited to visual restorations that do not impact performance, provided the product was initially functional. It is generally done when the end-user can accept a slight decrease in performance in exchange for a discounted price, and the supplier of the refurbished product opts not to fully remanufacture the product due to financial reasons or technical constraints.
- **Repurposing** is the process of reusing a product or its core(s) for a new application that differs from its originally intended use, without major reprocessing or remanufacturing. This is typically done when the product or component’s performance has degraded below the range necessary for its original application, and thus is not fit for remanufacturing or refurbishing, but can still be reused for a less demanding application. Repurposing for EV-LiBs can involve reusing EV-LiB modules or cells for energy storage or low-speed mobility, among other applications, where energy density is not a critical requirement, as is in automotive applications.
- **Recycling** is a process that recovers raw materials from EoL products, similar to those obtained from virgin resources. This recycled material is then processed again and used in the manufacture of new products. For EV-LiBs, recycling involves partial disassembly and shredding, followed by a pyrometallurgical or hydrometallurgical process to recover black mass, a powdered mixture of electrode materials. The black mass is further refined to separate the metals, which can be used for manufacturing. Ideally, recycling EV-LiBs should be considered a last resort, used only when cells or modules have no remaining useful life, meaning they are unsuitable for remanufacturing, refurbishment, or repurposing.

Based on personal correspondence with battery recyclers and remanufacturers in the United Kingdom (UK) and China, the implementation of this hierarchical circular strategy appears to be limited in practice. EoL EV-LiBs that can be processed are primarily recycled, and other streams of the hierarchical strategy are practised on a much smaller scale. As a result, a significant amount of residual value – the remaining economic value of an end-of-life (EoL) component or product that can be recovered through remanufacturing, refurbishing, or repurposing – is wasted. The remaining retired EV-LiBs, which constitute a significant portion of the total retired EV-LiBs, are stockpiled in warehouses, awaiting processing, due to insufficient infrastructure to support the broader implementation of the strategy [5].

A missing key component of the infrastructure is efficient, scalable, and cost-effective disassembly processes, which are essential for all streams as a first step [6]. Due to modern products not typically being manufactured for mass disassembly and the overall difficulty of automating disassembly, battery recyclers and remanufacturers must manually perform EV-LiB disassembly [7]. Manual disassembly is skill-intensive, hazardous [8], time-consuming, and prone to variability, making it difficult and costly to scale as volumes of EoL EV-LiBs increase, thereby constraining the throughput of remanufacturing, refurbishing, and repurposing processes [7]. The main challenges of robotic disassembly of battery modules include the removal of non-detachable joints, such as laser welding and adhesive joints, as well as battery hazards during disassembly and the lack of standards and regulations [9, 10]. It also requires a specialised workforce and substantial investment in training, making employers more exposed to fluctuations in the labour market, particularly given ongoing challenges in recruiting and retaining workers for physically demanding technical roles [11, 12]. Additionally, safety regulations often require a minimum spacing between workers, making floor space another limiting factor [13]. Therefore, automated disassembly processes are essential for unlocking the productivity of all streams within the hierarchical circular strategy for EoL EV-LiBs.

Recycling has become the most prominent option for processing EoL EV-LiBs, primarily because it requires only partial disassembly [2, 14, 15], thereby alleviating the labour requirements associated with manual disassembly. However, forgoing disassembly comes at the expense of increased processing costs and a greater environmental impact due to the additional energy required later to separate materials. Thompson et al. demonstrated that up to 80% cost savings can be achieved by using a recycling process that incorporates disassembly, compared to a process without disassembly [16], due to the prior separation of materials. Furthermore, bypassing disassembly reduces the purity of high-value materials recovered, limiting their use in products with stringent material requirements [17]. Therefore, improving disassembly processes is also desirable for recycling [18].

Given the clear need for automated disassembly, this case study presents a novel design for an automated disassembly process, specifically focused on module-to-cell disassembly of EV-LiBs. This focus is driven by the fact that, while recyclers and remanufacturers commonly carry out pack-to-module disassembly, they tend to avoid module-to-cell disassembly due to the increased complexity and safety risks associated with working closer to the cells [18]. As such, they are unable to implement more granular circular strategies, such as selective cell reuse or targeted remanufacturing, that could otherwise improve residual value recovery, reduce costs, and enhance revenue potential [17]. This has created strong motivation within the industry to automate the module-to-cell disassembly process. To ensure broad applicability, the proposed robotic disassembly cell is designed to accommodate EV-LiB modules from various manufacturers, which increases the value of the robotic cell to the end-user. The proposed robotic cell was developed with cost-effectiveness in mind; therefore, this study includes both a financial feasibility assessment and an evaluation of its functionality.

The paper’s main contributions can be summarised as follows:

- A prototype robotic cell for module-to-cell disassembly of an EV-LiB module with prismatic cells is designed and built. The machine is expected to be compatible with battery modules from various manufacturers.
- A disassembly process utilising the cell is designed to extract prismatic battery cells from an electric vehicle (EV) battery module. The disassembly process includes a mixture of destructive and non-destructive operations.
- This paper provides guidelines for designing a prototype robotic system for individuals seeking to develop similar systems for related purposes. It is strongly recommended that similar subsystems of this robotic cell be considered.
- The robotic disassembly system’s performance was assessed quantitatively and compared to that of manual disassembly processes.
- An evaluation method for the financial feasibility of the cell was developed and utilised to assess the economic impact of the proposed robot cell.

This paper is organised into the following sections. Section "Literature review" provides a brief literature review discussing previous related works to highlight the novelties of this research. Section "Battery module analysis" conducts a brief survey and analysis of battery module designs, identifies a class of battery module designs that share common characteristics, and formulates a generalisable automated disassembly sequence for that class of battery module design. Section "Physical robotic disassembly system" summarises the main aspects of the proposed robotic cell, describing its functions and how it performs the required disassembly operations. Section "Experiment" describes the experiment conducted to assess the execution time of the robotic disassembly process. Section "Results and discussion" compares the execution times of the robotic and manual disassembly processes.

## Literature review

Prior studies have presented work that contributes to the realisation of automated disassembly for EV-LiBs [6].

Some fundamental research [19–22] has focused on Disassembly Sequence Planning (DSP) [23, 24], which involves generating an optimal disassembly sequence given product information and a streamlined decision-making framework. These works aimed to apply and validate generalised DSP methodologies to EV-LiB module or pack disassembly, and to determine whether they generated more optimal sequences than intuition-based methods used by experienced technicians or other DSP methodologies. Because of this focus, these works did not focus on the realisation of a physical robotic disassembly cell to validate their sequences. Our work focuses more on the physical realisation and evaluation of a robotic cell design. The disassembly sequence used is not generated from a DSP method, but instead replicates the sequence already performed by skilled technicians.

Other fundamental research focuses on improving the robustness of robotised disassembly operations for generic robotic disassembly [25–27]. For instance, unscrewing [28–30] and object extraction [31, 32]. These works address the stochastic variability between instances of the same product [33], such as damage and condition, by deviating from traditional offline robot programming methods and developing online adaptive control algorithms that adjust to variability without further reprogramming. These works focus on validating their techniques on isolated tasks, rather than an entire disassembly process. Our work focuses on implementing an entire automated disassembly sequence specifically designed for EV-LiB modules and assessing its functionality and cost-effectiveness. However, our work does not implement advanced control strategies to improve the robustness of individual operations. This work assumes limited variability between product instances, so it utilises a rigid program that was generated offline; however, future research is intended to incorporate these techniques in later iterations.

Other works are more application-focused, proposing system designs for a robotised disassembly process [34–36]. Wegener et al. [37, 38] proposed a comprehensive robotic disassembly cell featuring Human-Robot Collaboration (HRC) for EV-LiB disassembly, from pack-to-module, then module-to-cell. Tan et al. [39] proposed a similar system, but only for pack-to-module disassembly. Fleischer et al. [40] analysed the equipment required for a robotic EV-LiB module-to-cell disassembly cell and proposed a design. Poschmann et al. [41] proposed a software architecture for an information-driven robotic disassembly. These works propose only concepts of a disassembly cell and still do not realise a physical robotic cell; thus, they cannot validate the functionality of the design nor assess the financial feasibility of such a machine, as in this work.

A paper by Qu et al. [42], from our group, presents a study closely resembling this paper, which proposed, built, and evaluated a robotic disassembly system. This disassembly system demonstrated the successful disassembly of EV-LiB packs into modules using non-destructive operations (e.g., unscrewing, pick-and-place, and cable manipulation). However, this previous study did not address the disassembly of modules into cells, as in this work. Additionally, our work introduces the use of destructive disassembly operations alongside non-destructive ones, providing a novel addition to prior work. Finally, this previous work also does not emphasise or demonstrate financial feasibility, whilst this work does.

Another work closely resembling this was presented by Choux et al. [43], who conducted an economic analysis assessing the financial feasibility of the automated disassembly of EV-LiBs from the module to the cell level. Their approach employed a probabilistic methodology to estimate the productivity of a hypothetical robotic disassembly line, which was then used to infer potential revenue gains across the full recycling pipeline. In contrast, this work offers a productivity analysis based on real operational data from a physical robot prototype. Furthermore, this paper’s economic analysis focuses on projecting cost savings relative to manual disassembly for the disassembly process only, and estimating the payback period for the capital cost of our proposed robot cell.

## Battery module analysis

This section presents an analysis of EV-LiB modules and how their characteristics informed the design of the proposed disassembly cell. It begins by outlining the rationale for favouring prismatic module formats, followed by an exploration of design trends across prismatic battery modules from various manufacturers. Common structural features are identified, which serve as the basis for developing a generalised disassembly approach applicable to a broad range of module designs. A standard set of disassembly operations is defined, along with a flexible disassembly sequence that can be adapted to accommodate design variations across different manufacturers.

### Suitability of prismatic EV-LiBs for automation

EV-LiBs are typically constructed from one of three main cell formats: cylindrical, pouch, and prismatic. These formats vary in shape, casing rigidity, and how they are assembled into modules and packs. Each format presents unique challenges for disassembly, particularly when it comes to automation. This study has chosen to concentrate on battery modules made up of prismatic cells for both strategic and technical reasons.

Prismatic formats currently dominate the global EV-LiB market. Recent data indicate that prismatic Cells accounted for approximately 69% of the global EV-LiB capacity in 2024 [44, 45]. As a result, EoL batteries entering the recycling and remanufacturing stream are predominantly prismatic, making them a prime candidate for automated disassembly.

From a technical perspective, prismatic modules offer several advantages for automated disassembly. They typically contain fewer, larger-format cells housed in rigid metal casings, which are easier for robotic systems to grasp, manipulate, and dismantle without damage. In contrast, cylindrical cells are small and numerous, requiring fine-scale manipulation, whereas pouch cells lack structural rigidity, making their manipulation with robots more challenging.

Recent research on robotic disassembly of EV batteries frequently focuses on prismatic modules due to these advantages. Industrial practitioners also exhibit this preference. Therefore, prismatic EV-LiB modules were chosen as the subject of this automation study.

### Prismatic battery module design survey

**Fig. 2:**
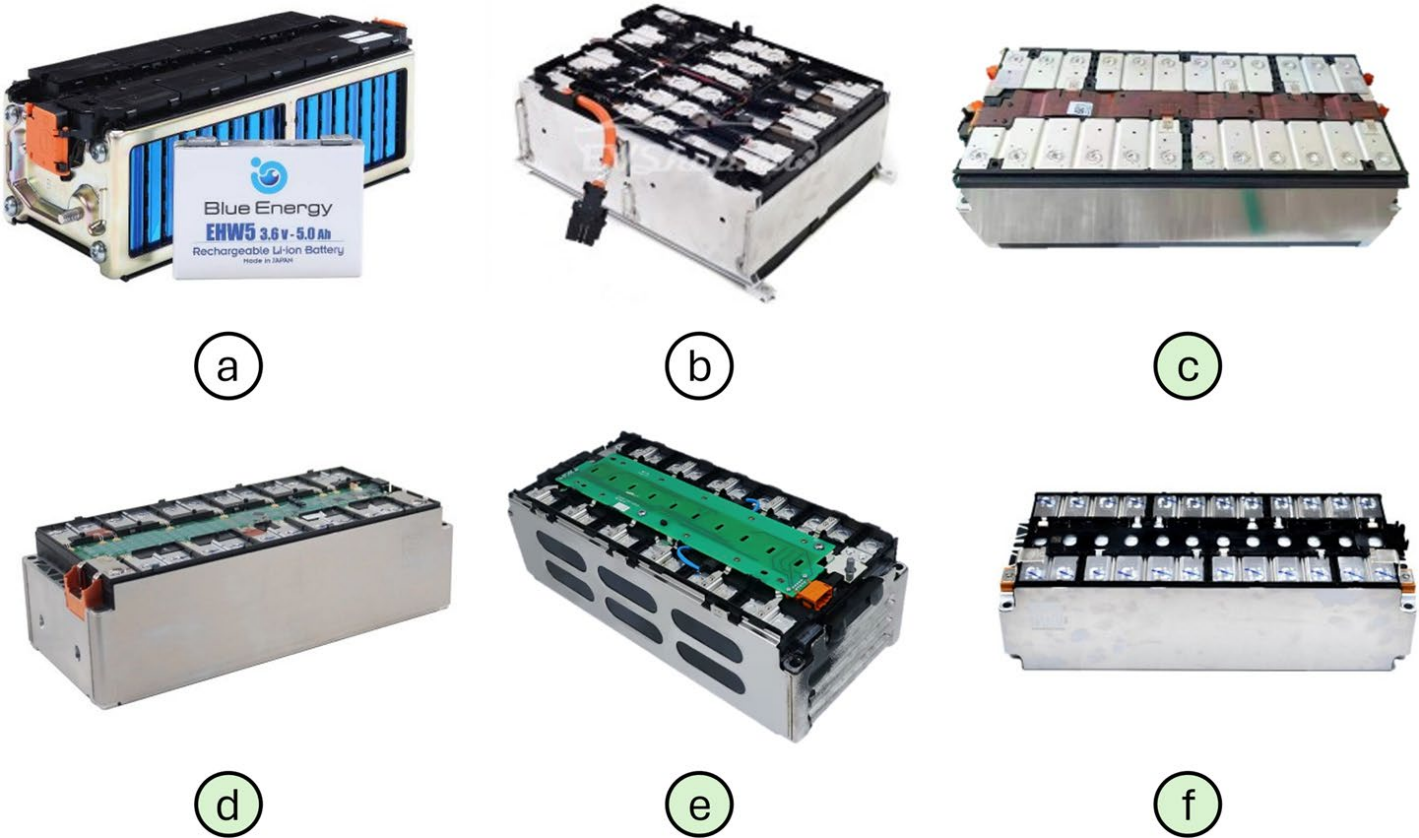
Images of popular battery module designs surveyed. **a** Blue Energy EHW5 [57], **b** BMW 12S1P [58], **c** CALB 4S3P [59], **d** CATL 6S1P [60], **e** Samsung 12S1P [61], **f** Stellanis 6S2P [62]. Green labels indicate that the corresponding module designs were selected to be compatible with the proposed robotic disassembly cell

A survey of battery module designs was conducted to observe structural commonalities between designs from various manufacturers. Table 1 presents the prismatic battery module designs considered in this study, and Fig. 2 illustrates their designs. Example vehicles were identified by searching for aftermarket spare part listings on various online commerce platforms [46–56].

**Table 1:** A table showing the EV-LiB module designs that were surveyed, with examples of EVs that use them listed alongside. Example vehicles could not be identified, as this module is used mainly in Chinese EVs, making it difficult to verify which models it was installed in.*

Battery Module Design	Dimensions (W x L x H) (mm)	Examples of Vehicles Using Them
Blue Energy EHW5	120 × 162.5 × 85	Honda Accord, Honda Freed, Honda Stepwgn Spada
BMW 12S1P	310 × 410 × 150	BMW I3
CALB 4S3P	151 × 355 × 108	N/a*
CATL 6S1P	151 × 355 × 108	MG ZS, MG Maxus, Nissan Leaf
Samsung 12S1P	151 × 355 × 108	Audi Q5, Audi Q7, Ford Kuga, Porsche Panamera, Range Rover Evoque, Volkswagen E-Golf, Volkswagen Golf GTE
Stellantis 6S2P	151 × 355 × 108	Opel Corsa E, Peugeot E-208

**Fig. 3:**
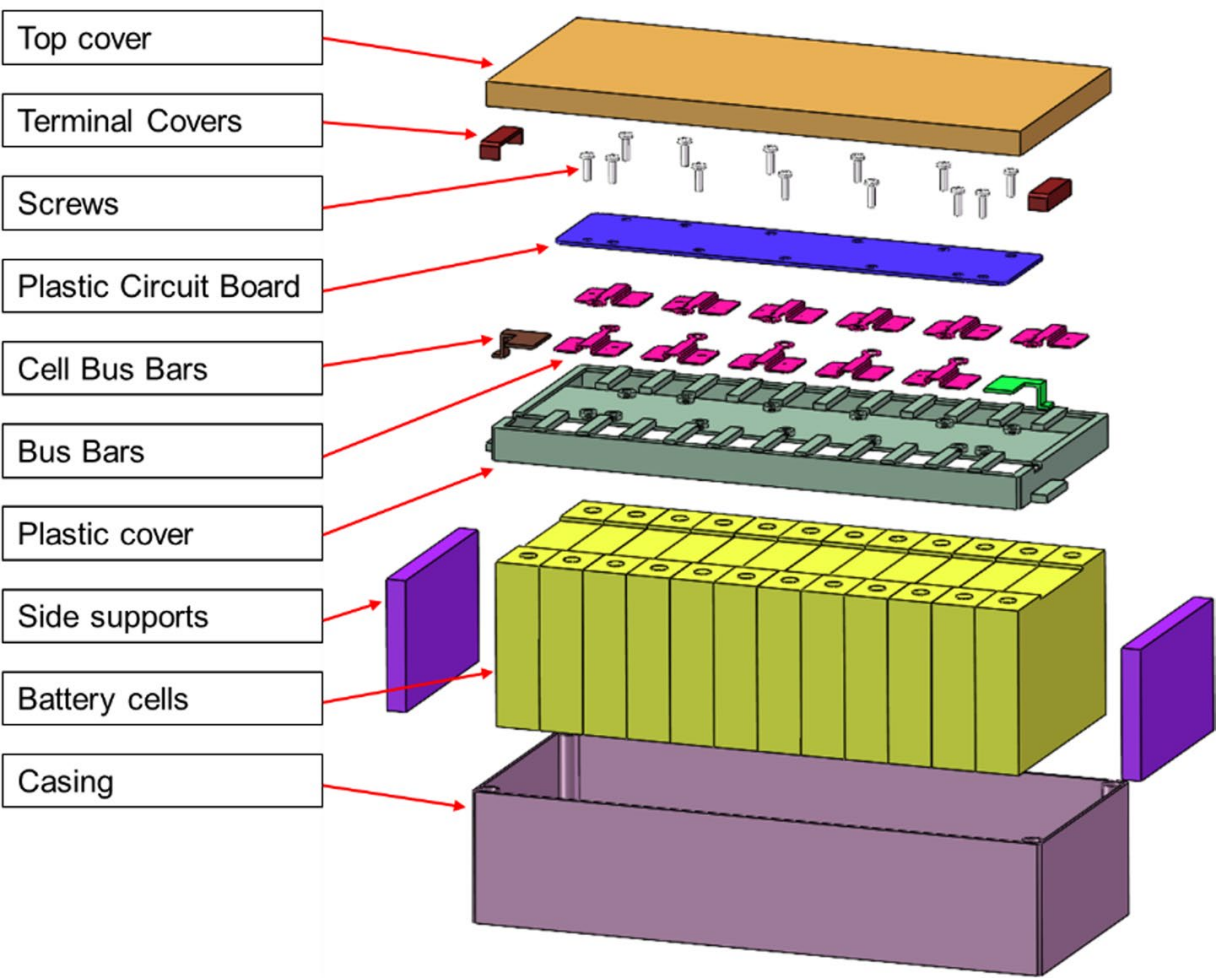
Exploded diagram of the Samsung 12S1P battery module, a design that adopts the VDA355 standard

From inspecting Fig. 2, commonalities exist among the majority of the surveyed EV-LiB modules. Except for the Blue Energy EHW5 (Fig. 2a) and BMW 12S1P (Fig. 2b), the remaining designs have the following similarities (Fig. 3):

- Prismatic cells are stacked horizontally in a single row with terminals facing upwards, forming a cuboidal shape. The cells are usually bonded together with adhesive to ensure no relative movement. The number of cells in each module design varies depending on the electrical rating of each cell and the desired rating of the overall cell stacks. It also depends on the thickness of the cells used. The module dimensions remain similar across designs as thicker cells are used when fewer cells are incorporated.
- A metallic casing wraps around the cell stack to provide protection and compresses the cells to minimise deformation during heat cycles. A metallic casing is used to enable heat to dissipate from the cells. The ends of the casing are welded together, forming a secure loop around the cells. It is fixed in place via a snap fit with the plastic cover. The casing also features through holes at each corner, which are used to fasten the modules to the battery pack.
- Cell terminals are connected with busbars, which are welded onto the cell terminals to ensure contact is maintained under vibration. The configuration of these busbars varies between designs. It can be based on the cell configuration, which is denoted in the name; for example, 12S1P means one set of twelve Cells in series, while 6S2P means two sets of six cells in series connected in parallel. It can also be based on the number of cells included in the module. The dimensions of these busbars differ slightly between manufacturers.
- A plastic cover is situated on top of the cell terminals, which the bus bars bridge over. This acts as an insulator between the terminals and bus bars, preventing unintended electrical contact between cells.
- A Printed Circuit Board (PCB) is placed on top of the plastic cover, which is part of the Battery Management System (BMS). These are connected to the busbars to collect voltage and current data from the cells, and a connector pin on the side of the module to transfer the data out of the module. The PCBs are either rigid, with Integrated Circuits (ICs), or flexible, without ICs. Rigid PCBs are used when a decentralised monitoring/processing unit of the BMS is placed within the module, if a distributed BMS configuration is used within the pack. These are fixed onto the plastic cover and bus bars with screws. Flexible PCBs are used simply to connect the busbars to the connector pins, which in turn are connected to the central BMS, if a centralised BMS configuration is used. These are fixed onto the plastic cover via snap joints and attached to the bus bars via soldering. The BMS configuration and inclusion of PCBs are driven by the EV pack’s design, in which they are installed.
- Finally, covers are installed over the top face of the battery module and the terminals of the cell stack for safety and to prevent unintended electrical connections. These covers are typically manufactured from plastic and attached via a snap joint. The geometry of the snap fit of the top cover is relatively uniform across designs from different manufacturers. The geometry of the snap fit on the terminal covers can vary between manufacturers and even vary between the same design from the same manufacturer. This variation is driven by the connection type required between modules by the EV manufacturer.

Upon further research, these commonalities were attributed to the adoption of VDA 355, a standard developed by the German Association of the Automotive Industry (abbreviated as VDA in German), which prescribes the structural characteristics as mentioned above [63, 64]. The suffix “355” refers to the prescribed module length being 355 mm [63]. VDA 355 was initially used as a standard within Volkswagen vehicles but was later adopted by other manufacturers, particularly in China, and utilised in a range of cars.

As the market’s energy demand evolved, the size specification of VDA Battery modules expanded, with variations such as VDA 590 and VDA 390, which feature the same general structure but differ in size [64]. An online marketplace [65] displays VDA Battery modules of various sizes, sourced from manufacturers such as CALB, CATL, Svolt, Tafel, and BYD. These modules resemble VDA 355 Battery modules and strongly indicate compatibility with a disassembly process targeted at a VDA 355 module.

This investigation suggests that numerous battery module designs possess common structural characteristics, which can be utilised to develop a generalised automated disassembly process for a substantial range of battery module designs.

### Disassembly sequence formulation

**Fig. 4:**
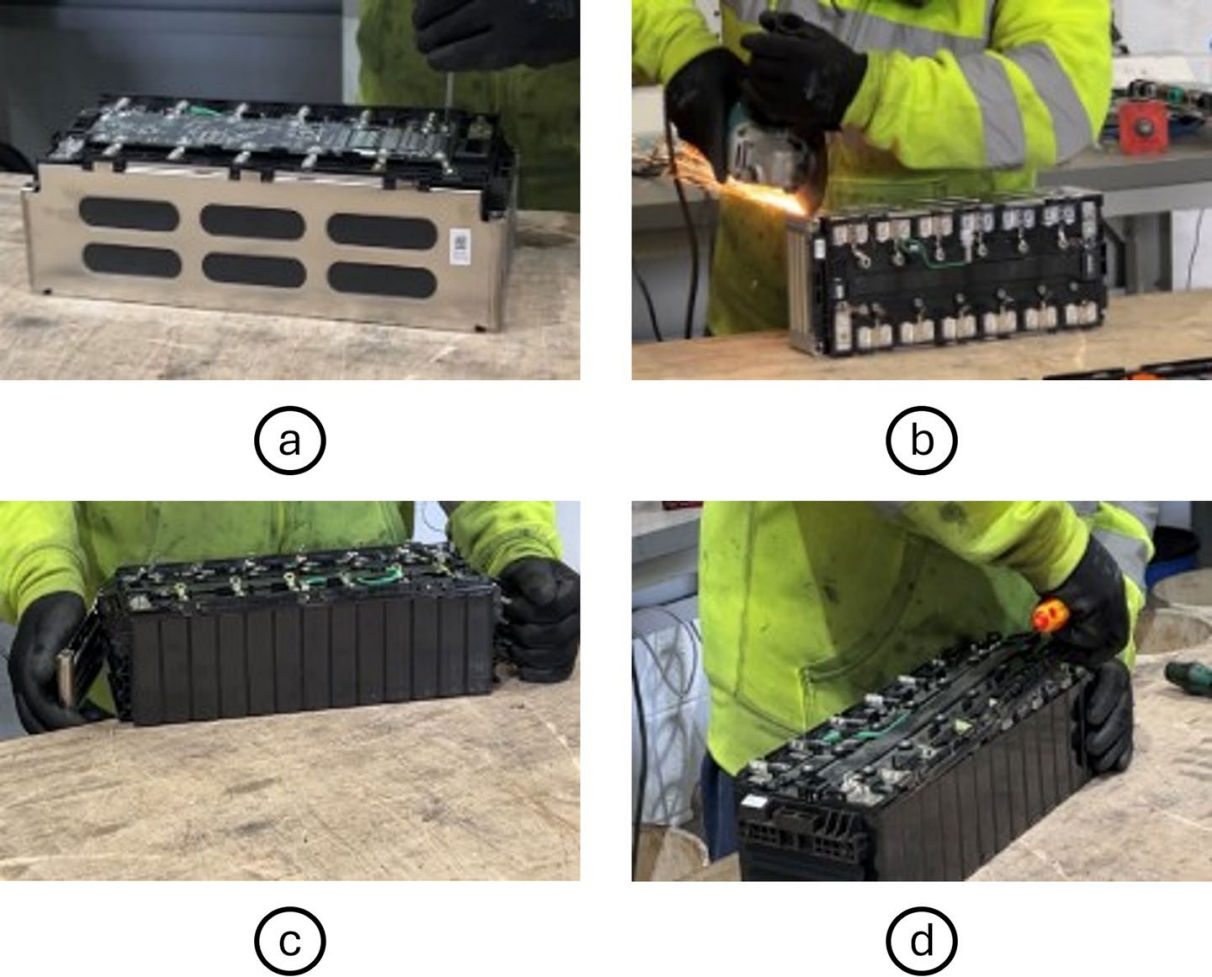
Images depicting an experienced technician performing the manual disassembly process. **a** Unscrewing task, **b** cutting along the height of the case at the corner, **c** bending open the case to reveal the cells inside, **d** prying off the plastic cover

To develop a disassembly sequence for implementation in the proposed robotic cell, the manual disassembly process of the Samsung 12S1P battery module, an example of a module conforming to the VDA355 standard, was used as a reference. The manual disassembly sequence is described below and is depicted in Fig. 4.

1. Prying task: remove the top cover and terminal covers.
2. Unscrewing task: Unfasten the screws that secure the PCB.
3. Pick and place task: Pick up the PCB off the plastic cover.
4. Cutting task: Cut along the middle of each busbar that connects the battery cells' terminals.
5. Cutting task: Cut the plastic cover along the length of the module near the edge on each side to disconnect the plastic cover from the casing.
6. Cutting task: Cut along the height of the module at the corner of the casing to disconnect it from the cell stack.
7. Bending task: Open the case, which is to be discarded.
8. Prying task: Remove the plastic cover
9. Prying Task: Wedge a prying tool between the cells to break the adhesive connection and separate them.

Table 4, in the appendix, presents the proposed automated disassembly based on the manual sequence, identifying the tools required for each task and considerations for generalising the automated process to other VDA Battery module designs. Steps 1, 8, and 9 (i.e., the prying tasks) were omitted from the automated process due to the low technical readiness of the technology required to automate those tasks. This is indicated by the fact that prior works that consider prying in disassembly studies always elect to perform prying manually [66–69], and no literature currently exists that studies robotic prying.

## Physical robotic disassembly system

**Fig. 5:**
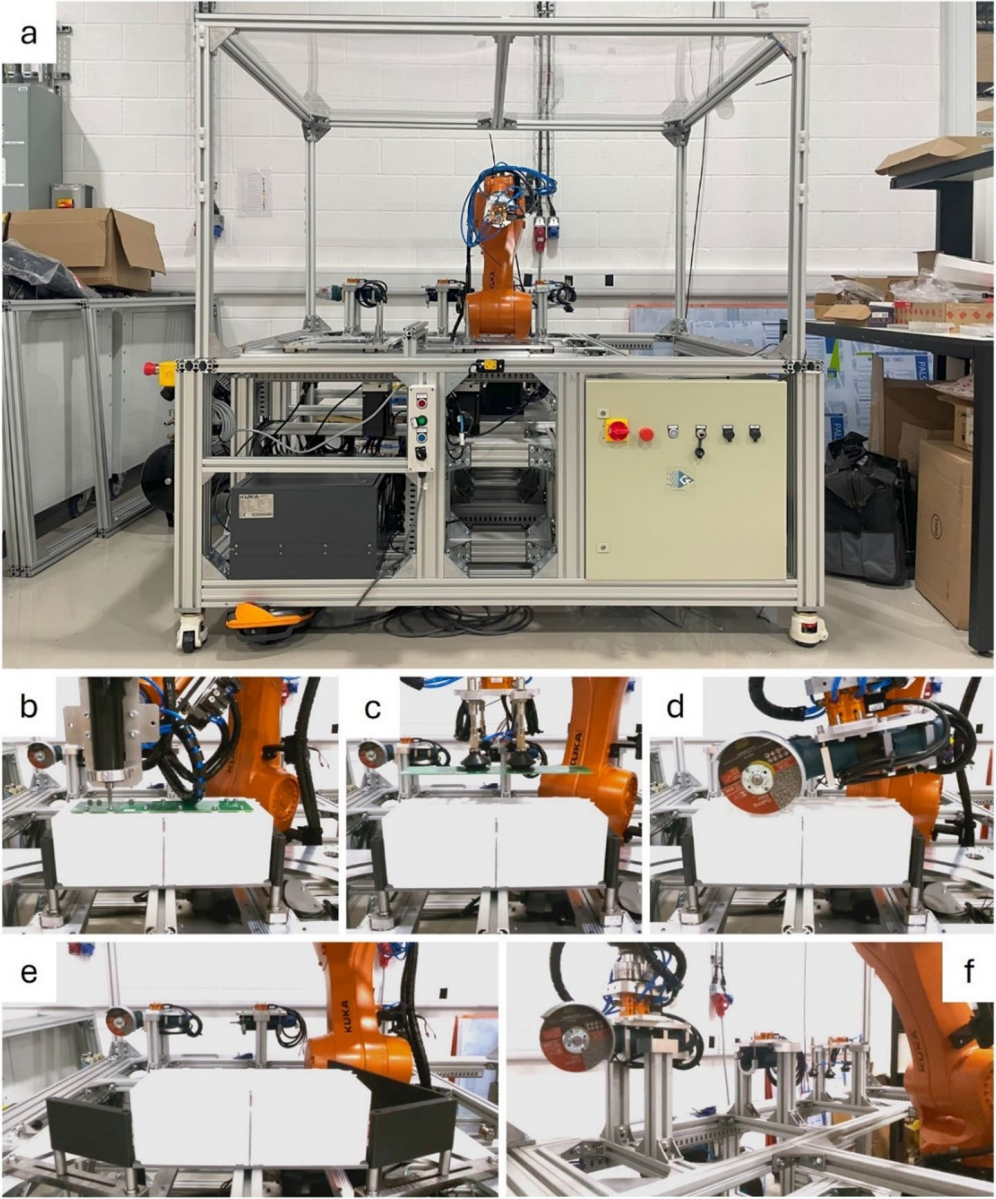
Robot cell for disassembling battery modules. **a** Overview of the real robot cell. Images of the robot cell performing the **b** unscrewing, **c** pick-and-place, **d** cutting, **e** bending, and **f** tool-changing tasks

A robotic cell to automate battery module disassembly was realised, as shown in Fig. 5, which implements the process detailed in Table 4. The main features of the robotic cell are a supporting structure, an opening tool system, electrical and electronic systems, a pneumatic system, and an industrial robot subsystem. This section will provide a brief overview of these key features in the following subsections.

### Supporting structure

**Fig. 6:**
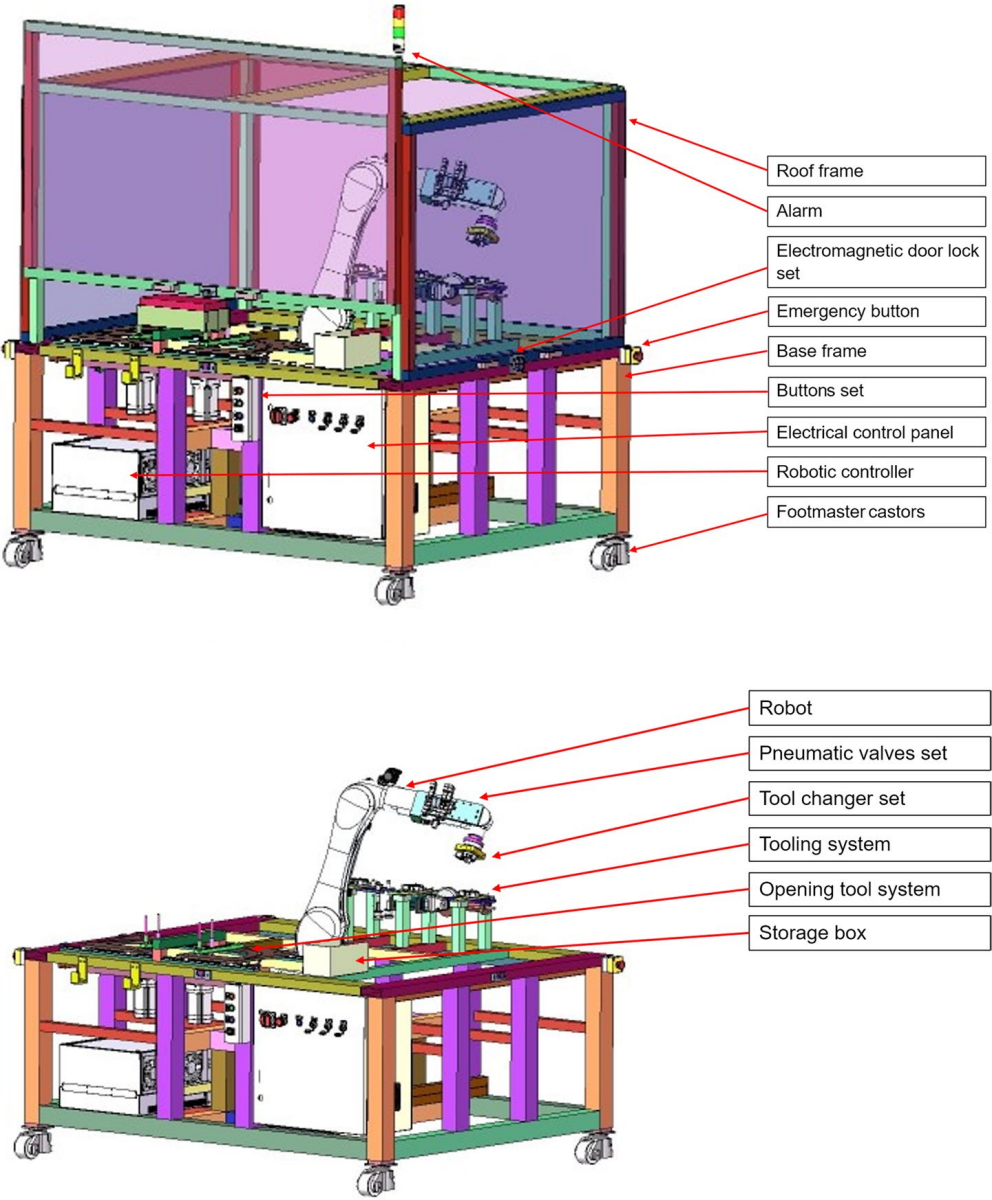
CAD model of the proposed robotic cell. The labels highlight the key components of the robot cell

The supporting structure system provides the main structure for the robot cell and houses all subsystems within it. The supporting structure comprises two sub-structures: the base frame and the roof frame (Fig. 6).

The base frame, assembled using industrial aluminium profiles and angle brackets, is the main structure supporting the robotic cell. Four height-adjustable footmaster castors were fastened to the bottom of the base frame, enabling the base’s position and height to be easily adjusted to provide flexibility for potential users with varying layout requirements. A storage box stores the components removed from the battery module.

The roof frame provides an enclosed working area for the robot, which contains hazards such as cutting debris, fires, and explosions, thereby enhancing safety within the robot cell. Four sliding windows on the roof frame provide convenient access to the cell. An electromagnetic door lock set serves as a safety lock, stopping the robot and tools when the windows are opened in automatic mode.

### Opening tool system

The tool opening system provides a fixture to secure the battery module in the cell and performs the bending task. A patent for this design has been submitted (application number GB2402201.4).

**Fig. 7:**
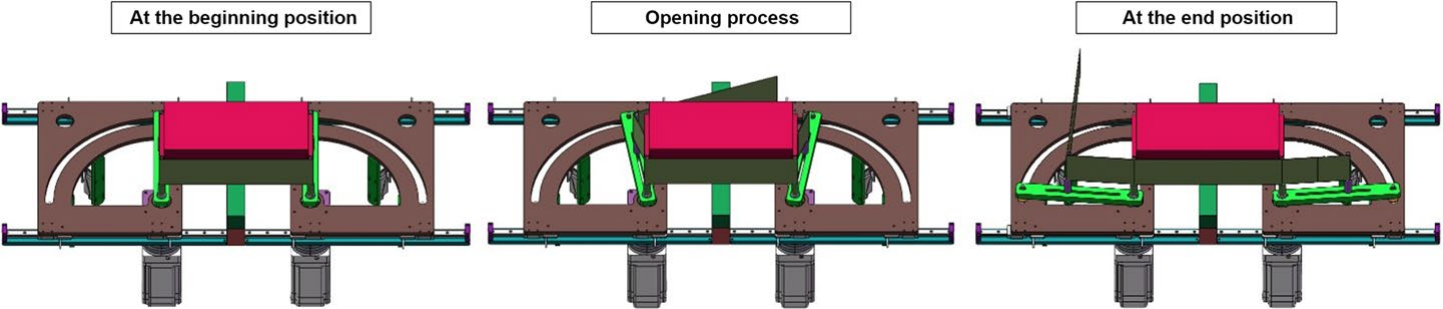
The opening tool system performing the bending task

The opening tool system is comprised of two symmetric modules fixed on guide rails, as shown in Fig. 7. In each symmetric module, there is a beam (coloured green in Fig. 7) with two pins protruding upwards that insert into two of the casing’s through holes from the bottom on each side. A motor, fixed underneath, pivots the beams from one end outwards, causing the case to bend and open after it has been cut, as shown in Fig. 7.

**Fig. 8:**
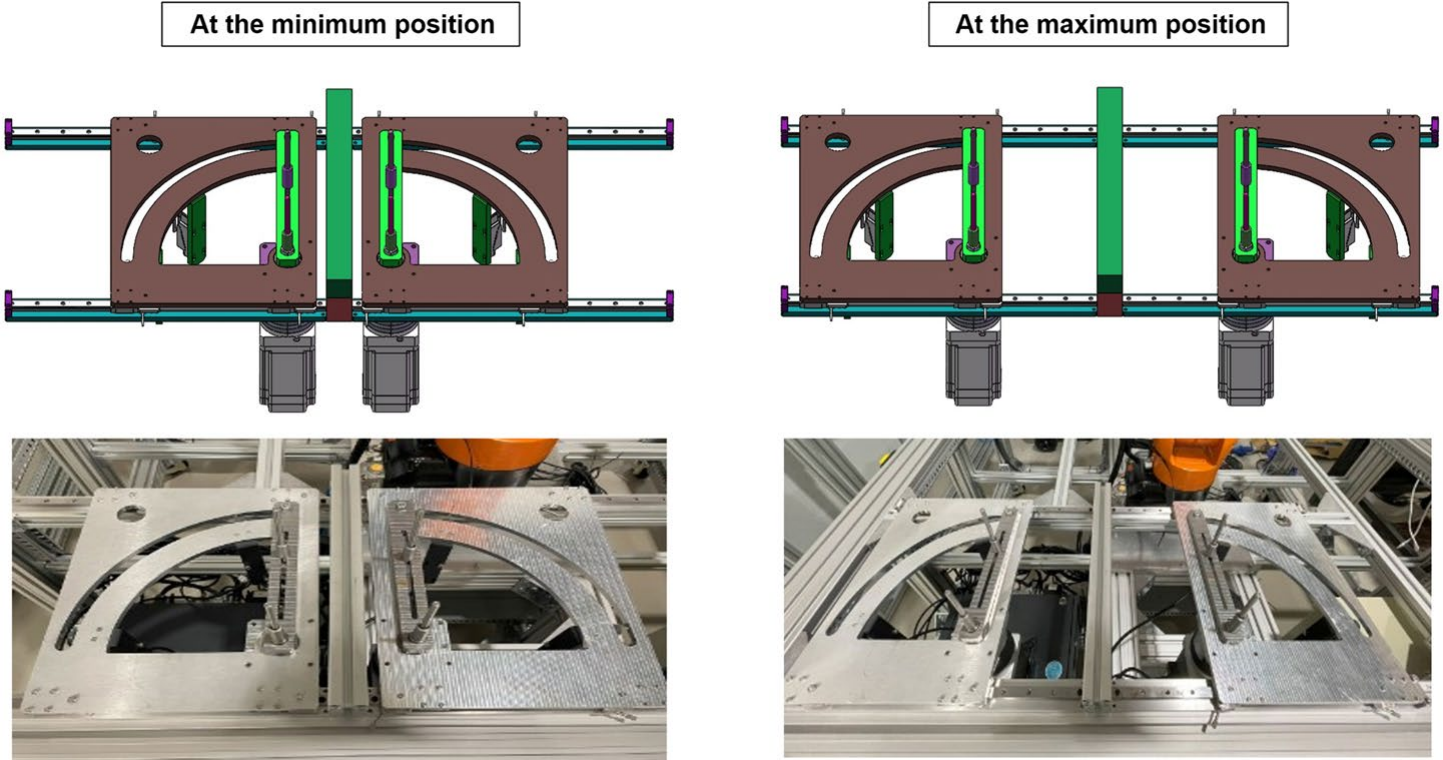
Images illustrating the adjustability of the opening tool system

To accommodate different battery module sizes, the fixture width and breadth can be adjusted. To adjust the width (left-right in Fig. 8), each of the symmetric modules can be slid along the guide rails and locked in place with rail locks underneath each plate. To adjust the breadth (up-down in Fig. 8), the distal pin in each beam can move freely along the beam’s long axis. This feature accommodates batteries sized between 140 and 450 mm in the X direction and between 36 and 195 mm in the Y direction, making it compatible with VDA 355 and VDA 390 Battery modules but not the larger VDA 590 modules.

The battery module is placed onto the opening tool system by manually inserting four pins protruding out of the top of each beam through the four holes at the corners of the battery module from underneath (Fig. 7). The module is fixed using friction, achieved by rotating the beams outward slightly to push the pins into the inner walls of the casing’s through holes.

### Tooling system

**Fig. 9:**
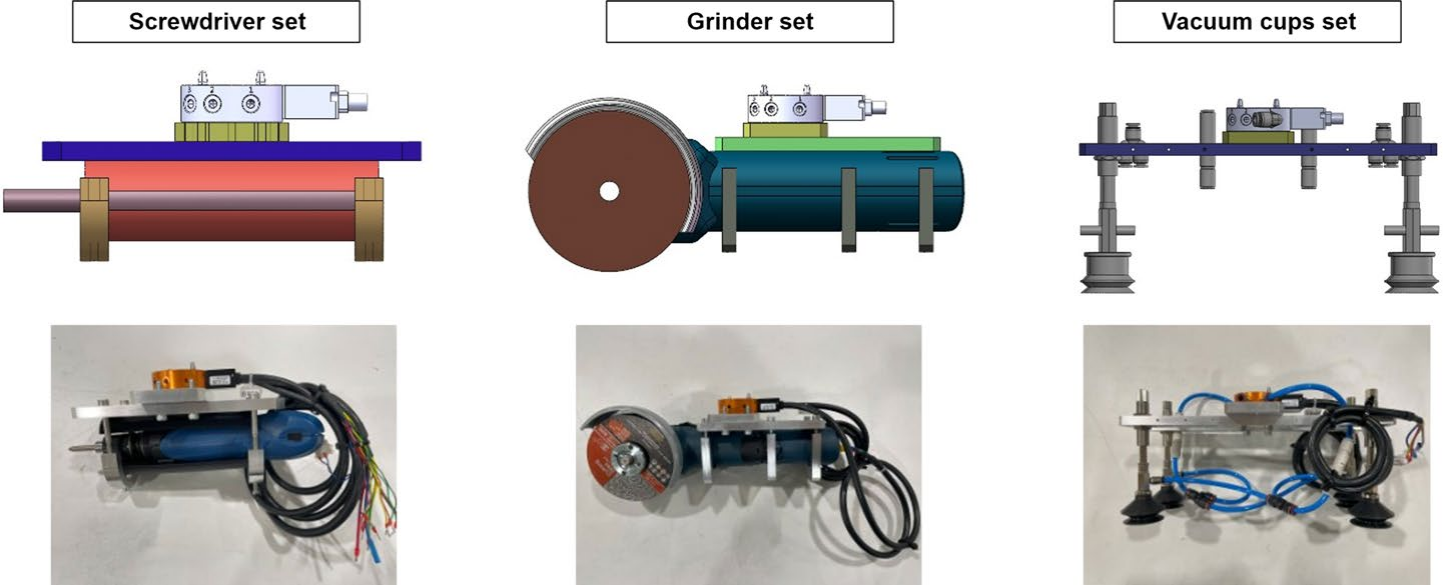
Images of the three toolsets included in the tooling system. Each column shows a CAD model and an actual image of each toolset. Each toolset comprises the tool and the female tool changer attached to it

The tooling system comprises three distinct tool sets: a screwdriver set, a grinder set, and a vacuum cup set, each designed to address specific tasks, including unscrewing, cutting, and pick-and-place (Fig. 9). Tool selection should consider (a) task requirements, force and torque required e.g., (b) cost effectiveness and (c) safety. The screwdriver and the grinder are modified from manual hand-held tools. The manual screwdriver with adjustable torque mechanism is a low-cost solution than industrial robotic screwdriver. In the Unscrewing task, it only required to remove M5 cross screws, thus, the automatic bit changing does not considered in the design to reduce the cost. The most safety issues of cutting has been considered in grinder design as an mature industrial product. Thus, the screwdriver and the grinder are modified from manual hand-held tools.

Each tool set has a female tool changer (Linghang LTC-0010 F). The male tool changer attached to the robot can securely attach to different female tool changers, facilitating the seamless interchangeability of multiple tools. The tool changer also facilitates the transfer of air and electrical current between the male and female tool changers. Electrical current activates the screwdriver and the grinder. Air generates a vacuum for the vacuum cup, enabling it to pick up objects. The transfer of air and electrical current is toggled using a controller.

Automated bit changing would be ideal to accommodate different screw drive types and sizes for various battery module designs, as stated in Table 4, without manual intervention. However, for this case study, automated bit changing was not implemented; instead, it relies on manual bit changing for now. This will be addressed in future works.

### Electrical and electronic systems

**Fig. 10:**
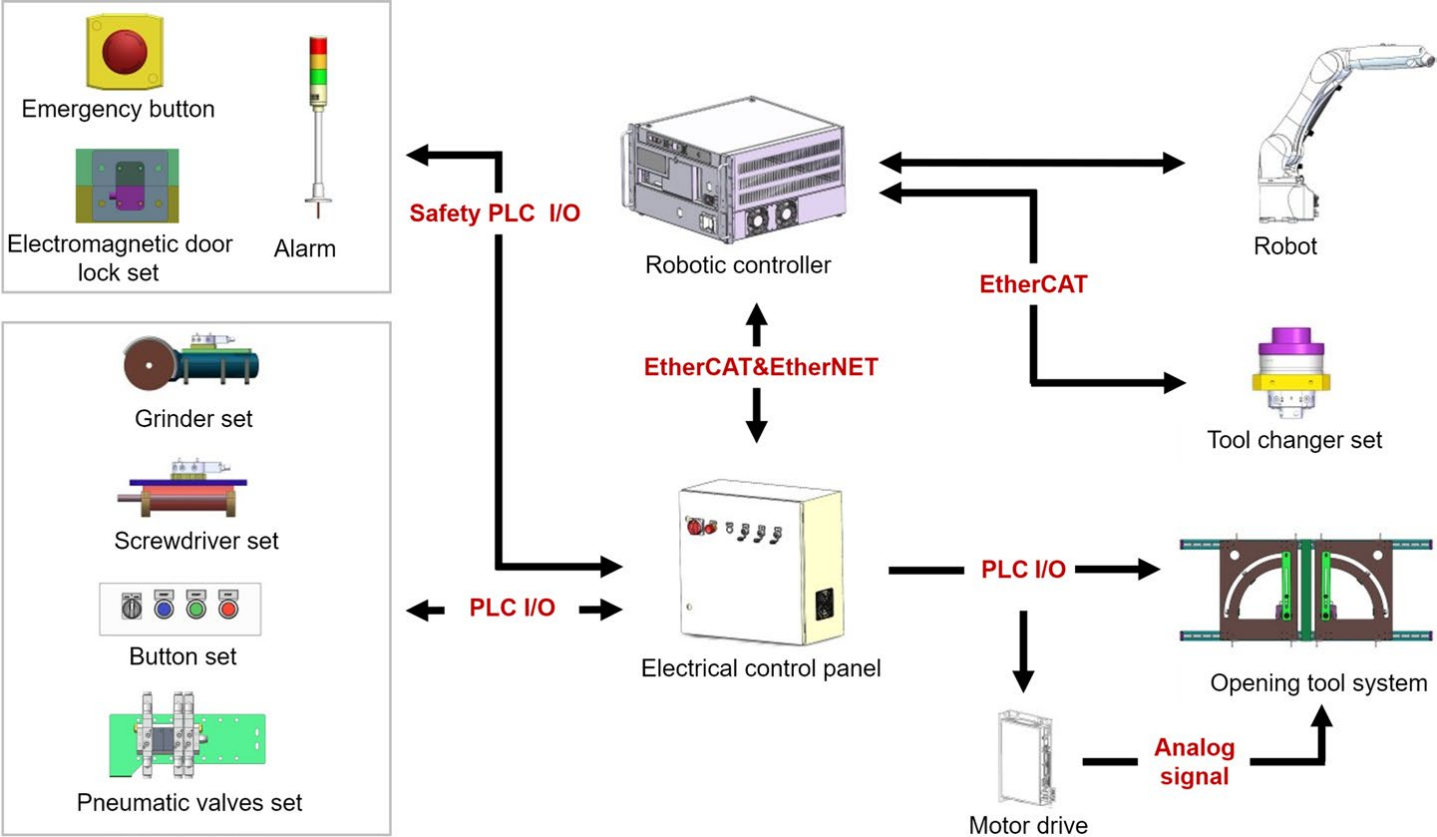
Electrical and electronic system overview. The arrows indicate the power transmission and information exchange between components, illustrating their relationships

The electrical system provides power to the tool opening system, tooling system, information communication platform, and safety monitoring system. The electrical system provides electric power to this cell’s actuators, sensors, communications systems, control systems, and safety functions (Fig. 10). The electronic system comprises a control and communications system, as well as a human-machine interface to the robot cell.

The human-machine interface of the robot cell consists of a button set and an emergency button, which are fastened to the base frame. The button set features a start, stop, and reset button, as well as a switch to toggle between manual and automatic modes for cell operation. The test mode is intended for debugging and testing, allowing the system to control tools when the windows are opened. The test mode also limits the robot’s maximum speed to ensure safety. The automatic mode is designed to run the robot while monitoring the safety system and can operate at a maximum speed four times that of the maximum speed in test mode.

The control and communications system comprises a robotic control unit and an electrical control panel, which are fastened to the base frame. The robotic controller (Kuka Compact 4) controls the robot’s movement and facilitates communication with the Force/Torque (F/T) sensor on the tool changer set. The FT sensor provides force and torque information for precise robot control in the Unscrewing and Cutting tasks. The electrical control panel serves multiple functionalities:

i) It provides a software platform for programming and execution to control components via an industrial PC (Beckhoff C6920).
ii) It facilitates information exchange between components (industrial PC, robotic controller, and all tools) via a coupler (Beckhoff EK1100 and EK1110) equipped with a series of Input/Output (I/O) terminals.
iii) It separates non-safety (controlling tools, opening tools, system actuators, and robot) and safety (monitoring safety conditions of the cell) I/O communications to ensure that the robot cell operation can terminate in an emergency.

The non-safety I/O terminals (Beckhoff EL1859, EL1819, EL2809) connect to the tooling system and pneumatic valves, which are set to issue binary start/stop commands to these systems. They also connect to the motors and position switches in the opening tool system, facilitating the actuation of the motors. Furthermore, the I/O terminals are connected to the button set.

The safety I/O terminals (Beckhoff EL1904 and EL2904) connect to the alarm, electromagnetic door lock set, and emergency button. The alarm indicates whether the robotic cell is inactive, active, or in emergency stop mode. The electromagnetic door lock set only operates in automatic mode, which stops tool and robot operation when disengaged by lifting the windows.

### Pneumatic system

**Fig. 11:**
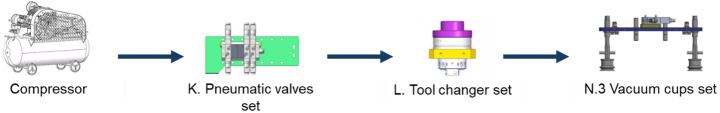
Pneumatic system overview. The compressor provides compressed air, which passes through the pneumatic valve set and tool changer set before reaching the vacuum cup set. The vacuum cup set utilises compressed air to create a vacuum, allowing it to lift objects with the aid of suction force

The pneumatic system has two functionalities, including locking or unlocking the tool changer set and generating a vacuum for the vacuum cups to perform the pick-and-place task (Fig. 11). A compressor supplies air to the pneumatic system and maintains a pressure of 0.7 MPa in the loop. The air passes through the pneumatic valve set via a combination filter.

### Industrial robot system

The robot is an industrial robot (Kuka KR10 R1100) bolted to the base frame. The pneumatic valve set, attached to the 5th joint of the robot, controls the air input to various subcomponents of the robot. The tool changer subassembly, fastened to the end effector of the robot, includes an F/T sensor (ATI Axia80-M50 EtherCAT) and a male tool changer (Linghang LTC-0010FM). The F/T sensor measures forces exerted on the robot’s end-effector during robotic disassembly. Attached to each tool is the female tool changer, which mates and locks/unlocks with the male tool changer to facilitate tool changing.

**Fig. 12:**
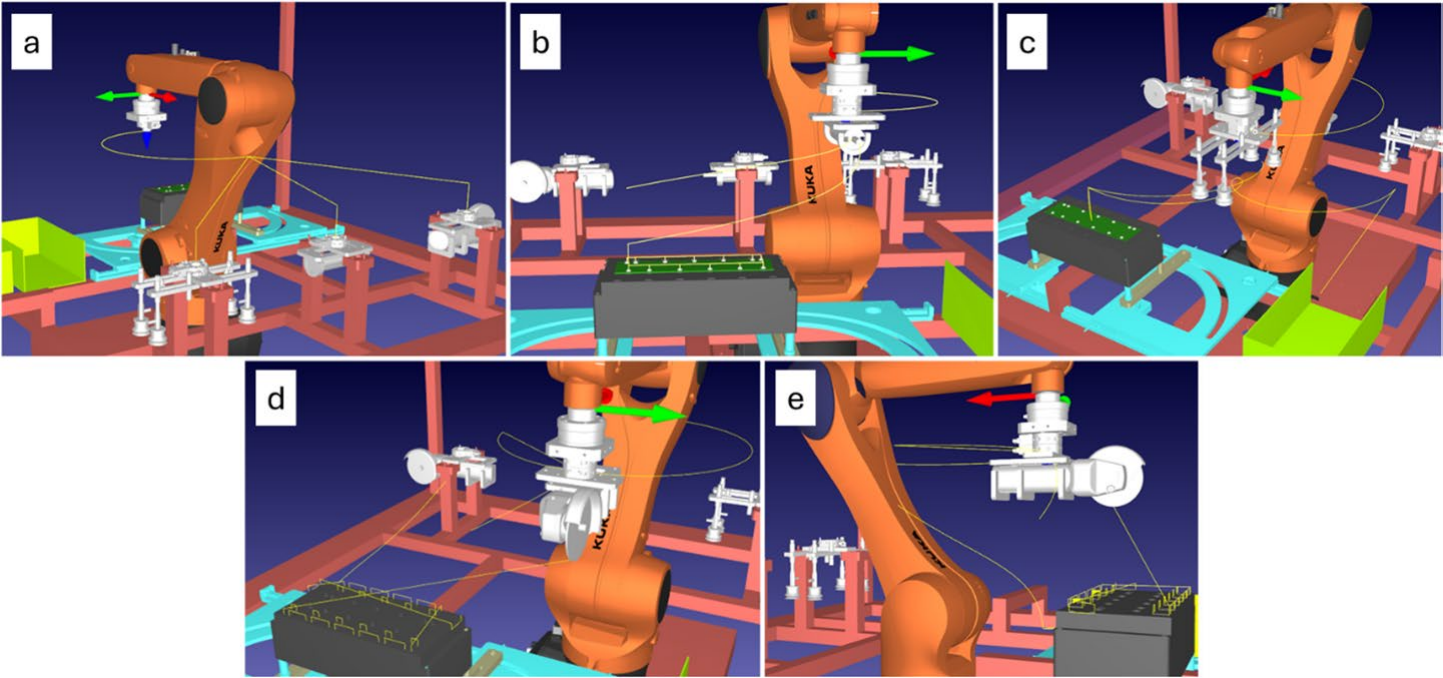
Images depicting the robot’s end effector’s paths in the programmed sub-tasks. **a** Tool changes, **b** unscrewing task, **c** pick and place task, **d** and **e **cutting task. These images were taken from a simulated robot model implemented in commercially available software (RoboDK v5.6.8)

The robot executes the motions required for each operation. For ease of re-programmability, the robot program is constructed using movement primitives, which are parameterised sub-routines that can be reused and easily reconfigured based on the specification of a battery module. The movement primitives, the tasks they are used in, and the parameters that can be configured are listed in Table 5 in the appendix. Images of the movement primitives being executed are shown in Fig. 12. The movement primitives were programmed using an offline programming approach, so they do not offer that much robustness if the parameters are not set precisely. However, this will be addressed in future work.

## Experiments

To validate the proposed robot cell, its productivity and cost-effectiveness were assessed in comparison to its manual counterpart. By comparing the cost and productivity, the potential savings and payback period of implementing the robotic cell could be evaluated, highlighting the economic benefits of the investment.

In the notation used below, each of the symbols introduced may later be accompanied by an $\:auto$ or $\:man$ to indicate that the Quantity is related to the automated or manual disassembly process, respectively. In these experiments, the Samsung 12S1P module was used as an example of a VDA standard prismatic battery module, which belongs to the class of battery modules that the proposed robot cell will be compatible with.

### Productivity analysis

Productivity was evaluated by first measuring the execution times ($\:T)$. $\:{T}_{auto}$ is the execution time using the real robot (Fig. 5), executing the program shown in Fig. 12 and at speeds shown in Table 6 in the appendix.

During the experiment, the robot’s speed was restricted to 250 mm/s (test mode limitation), which is roughly four times slower than its actual operating speed. Therefore, the measured times were divided by a timescale factor of four to reflect expected execution times in actual operational conditions. The experiments were also conducted on a mock-up model of the battery module to eliminate fire and explosion hazards, given the limitations of the testing facilities. The mock-up battery represents all features of the selected battery models, including screws, PCB, cutting features, and the case. Thus, the disassembly processes of the mock-up battery are identical to those of the real battery. $\:{T}_{man}$ is the time taken by a human operator to disassemble a real battery module.

For the productivity analysis, the weekly productivity rate (expected battery modules processed each week), $\:P$, was used as the metric to quantify productivity and was calculated as shown in (1). $\:D$ denoted the number of operating days per week, $\:H$ is the number of operating hours per week, and $\:{T}_{down}$ is the downtime between battery modules expressed in seconds. Table 2 shows the assumed parameter values for $\:D$, $\:H$ and $\:{T}_{down}$.

1 $$\mathrm P=\:\mathrm D\cdot\mathrm H\cdot\frac{3600}{\mathrm T+{\mathrm T}_{\mathrm{down}}}$$

**Table 2:** Weekly productivity analysis for the robotic disassembly cell and a human worker

Category	Parameters	Automated	Manual
Working Hours	Hours Working Per Day, $\:H$	24.00	7.50
	Days worked per week, $\:D$	5.00	5.00
Disassembly Time	Disassembly Time (s), $\:T$	223.75	601.00
	Downtime Between Modules (s), $\:{T}_{down}$	300.00	300.00
	Total Time per module (s)	523.75	901.00
Total Productivity	Total Modules per Week per Unit, $\:P$	824.82	149.83

### Economic analysis

The weekly operating costs, $\:C$, of the automated and manual processes were compared to determine the economic impact of the robot cell. In the cost analysis, only energy ($\:{C}_{e})$ and labour ($\:{C}_{l}$) costs were adopted using estimates from online sources [70–73]. To establish a baseline for comparison, the costs of the automated process were evaluated against those of a manual disassembly performed by human operators that matched the robot cell’s weekly productivity. The number of workers, $\:N$, for the manual process was determined using (2).

2 $$\mathrm N=\frac{{\mathrm P}_{\mathrm{auto}}}{{\mathrm P}_{\mathrm{man}}}\:$$

Table 3 summarises the assumed costs for the automated and manual disassembly operations. The individual costs presented in the table are detailed below.

**Table 3:** Expense analysis for a disassembly operation with and without a robot cell. The costs are for an operation targeting 824.82 battery cell modules processed weekly, which is the expected productivity of one robot cell (Table 2)

Category	Parameters	Automated	Manual
Worker Costs, $\:{C}_{l}$	Disassembly Worker Annual Weekly Salary + Overhead [31–33]	£ 1,021.09	£ 1,021.09
	Maintenance Worker Annual Weekly Salary + Overhead [31–33]	£ 1,021.09	-
	Total Worker Weekly Costs	£ 2,042.19	£ 1,021.09
Energy Costs, $\:{C}_{l}$	Energy Required to Power Robot Cell (kW)	3.68	0.00
	Daily Robot Cell Power Consumption (kWh)	88.32	0.00
	Energy Unit Price, UK (£/kWh) [34]	£ 0.25	-
	Daily Energy Cost	£ 21.64	-
	Weekly Energy Cost	£ 108.19	-
Total Weekly Costs, $\:{C}_{l}$	No. Units Need to Process 824.82 Modules/Week	1.00	5.50
	Total Expenses per Week per Unit	£ 2,150.38	£ 1,021.09
	Total Expenses per Week	£ 2,150.38	£ 5,621.04

For the automated process, it was assumed that one worker would be required to perform the prying and battery cell/side support separation tasks, and one worker would maintain the robot cell. The maintenance worker would be responsible for physical maintenance of the system, as well as reprogramming if the robot program required re-parametrisation for new battery module designs (Table 5). The total cost per worker is assumed to be £53,096.88, which is the median salary of a skilled engineering technician (£32,675.00) [70, 71] multiplied by the average overhead multiplier per worker (1.625) [72]. For a weekly rate, this would correspond to £1021.09 per worker. For the energy costs, it was assumed that the robot operates at 3.68 kW (32 A at 230 V) with an energy unit price of £0.245/kWh [73]. The robot operates 24 h a day and five days a week, so the weekly energy costs equate to £108.19.

For an operation using only human operators with the same productivity as one robot cell, $\:N$ human operators are required. Each operator will have a weekly rate of £1021.09, the same as the workers in an operation with a robot cell. The overhead multiplier was also assumed to cover the energy costs incurred by humans using power tools for manual disassembly, so no additional energy expenses were considered for the human-only disassembly operation.

By obtaining $\:{C}_{l}$ and $\:{C}_{e}$ for the manual and automated operations, the potential weekly savings from using the robot cell ($\:S$) can be calculated as shown in (3). This highlights the cost reduction from adopting the proposed robot cell in a disassembly operation every week.

3 $$\mathrm S={\mathrm C}_{\mathrm{man}}-{\mathrm C}_{\mathrm{auto}}=\mathrm N\cdot\left({\mathrm C}_{\mathrm l,\mathrm{man}}+{\mathrm C}_{\mathrm e,\mathrm{man}}\right)-({\mathrm C}_{\mathrm l,\mathrm{auto}}+{\mathrm C}_{\mathrm e,\mathrm{auto}})$$

Then, the calculation of the payback period ($\:{T}_{payback}$), the number of years until the cost of the initial investment ($\:Q$) has been regained through a discounted cash flow, which can be calculated using (4). This metric indicates the time required for a customer to recover their initial investment and is used to assess the feasibility of an asset investment [74].

4 $${\mathrm T}_{\mathrm{payback}}=\frac{\mathrm Q}{52\cdot\mathrm S}$$

## Results and discussion

**Fig. 13:**
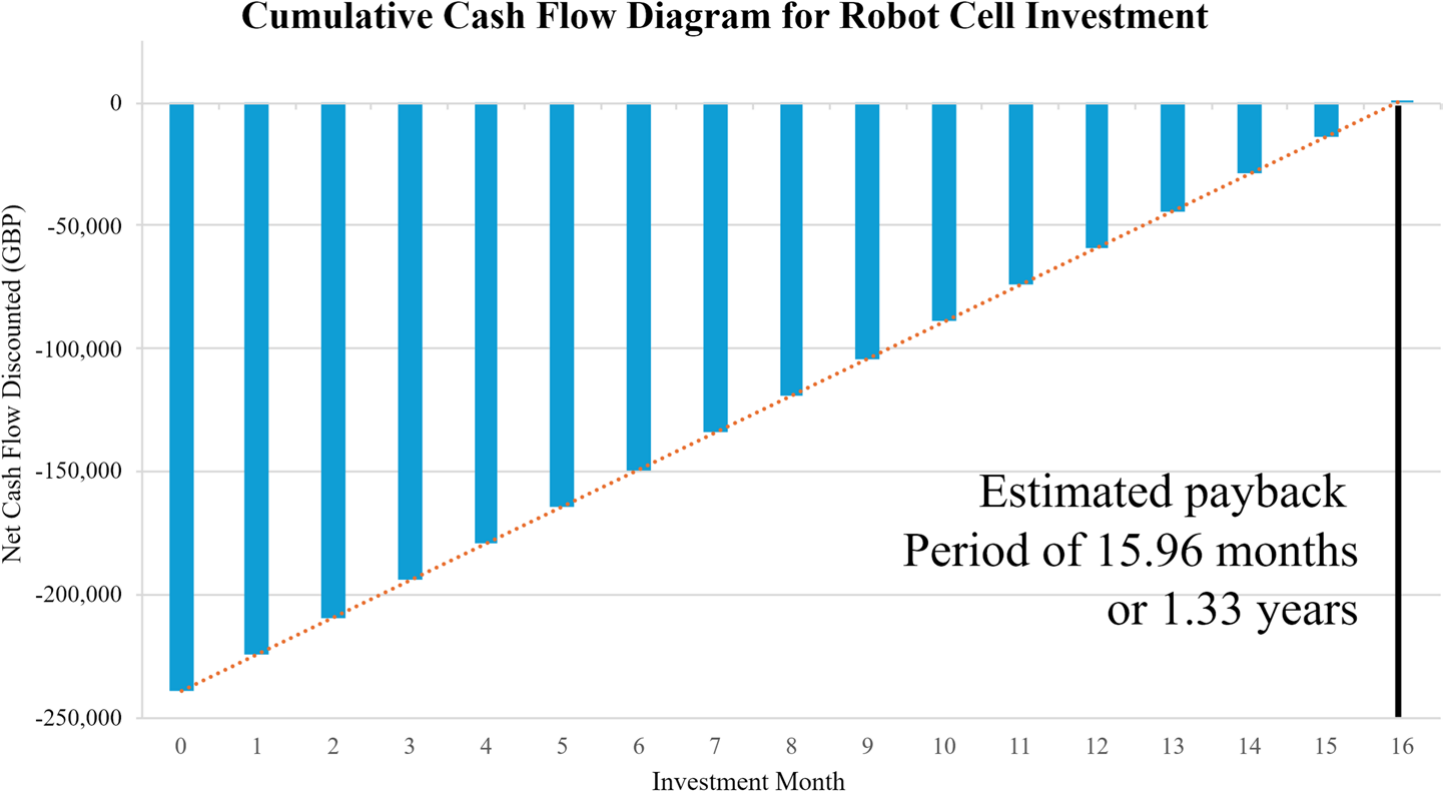
Cumulative cash flow diagram illustrating the payback period for implementing a robot cell to replace 5.50 workers in EV battery module disassembly

This section presents the results from the productivity and economic analysis. Table 2 illustrates the potential productivity increase that can be achieved by adopting a robotic disassembly cell. Table 3 compares the expected monthly expenses between robot and human-only disassembly operations. Figure 13 illustrates the expected payback period of the robot cell. The video demonstrating a fully robotic operation on a 3D-printed dummy of the selected battery module is available on this link [75].

### Productivity analysis

**Fig. 14:**
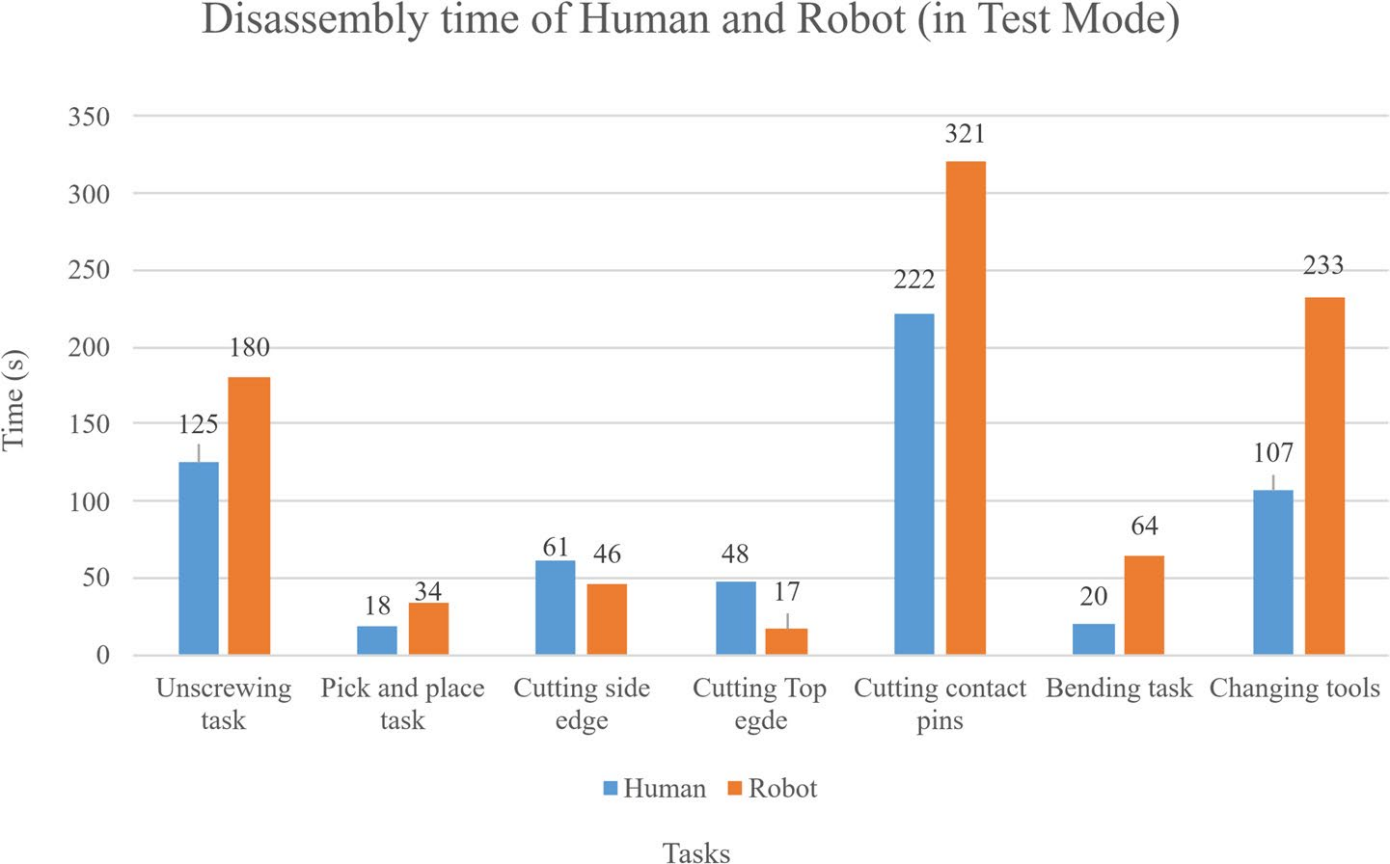
Disassembly time comparison between robotic (in test mode) and manual processes, subdivided into sub-tasks

Figure 14 compares the measured execution times of the automated and manual disassembly processes. The manual process was measured from one disassembly trial performed by a skilled worker at a local recycler. Overall, robotic disassembly was completed in 895.00 s in test mode, and an experienced technician completed the disassembly in an average of 601.00 s (Fig. 14). However, considering the robot cell will be four times faster in actual operating conditions, robotic disassembly can be expected to finish in 223.75 s, equating to a 168.60% increase in speed compared to manual disassembly.

Based on the assumed parameter values presented in Table 2 and using (1), the number of Battery modules processed per week for the robot and human was calculated to be 824.82 and 149.83, respectively. The significant increase in the robot cell’s productivity resulted from faster disassembly time and longer operating hours.

### Economic analysis

Using (2), the number of human workers in a manual disassembly operation that can match the productivity of the automated process was found to be 5.50 workers. Using the assumed costs presented in Table 3, the weekly expenses for the robot and human-only operations summed up to £2,150.38 and £5,621.04, respectively. Using (3), a significant potential weekly savings ($\:S$) of £3,470.66 can be expected upon adopting the proposed robot cell.

Based on the robot cell’s material and manufacturing costs and a profit margin of 100%, it is expected that the robot cell’s retail price ($\:Q$) to be £239,490.82. The retail price is calculated based on the labour cost for development, the hardware and software costs of the robotic cell, and a suitable profit margin. Based on values for $\:Q$ and $\:S$ and using (4), the expected payback period ($\:{T}_{payback}$) for the proposed robot cell could be as short as 1.33 years (Fig. 13), considered below average compared to the reported average investment payback period of 2.91 years in the US [74].

## Conclusion

A robotic cell for automated disassembly was proposed to address the safety and cost-effectiveness concerns of manual EV battery module disassembly. The key aspects of the robot cell’s design were discussed, including the industrial robot, the supporting structure, the tool opening system, the electrical and electronic system, and the pneumatic system (Section "Physical robotic disassembly system"). The robot cell design incorporates features that enable flexibility for different battery module designs following the VDA battery module standard, allowing for future processing of battery modules from various manufacturers with minimal reconfiguration. The design also features safety features, including an enclosed workspace to contain hazards (Section "Supporting structure") and a safety monitoring system that terminates the cell’s operation when its windows are opened and the emergency stop button is pressed (Section "Electrical and electronic systems").

To demonstrate the practicality of this robot cell, the cost-effectiveness was assessed by comparing the productivity and cost of the automated process with the manual process (Section "Results and discussion"). The initial assessment yielded promising results, with the robot cell demonstrating the same productivity as 5.50 workers within a standard workweek, resulting in £15,039.53 in expected monthly savings. Should an organisation replace 5.50 workers with a single robotic cell, a payback period of 1.33 years is expected.

The automated disassembly process was tested on a mock-up model of the Samsung 12S1P battery module to ensure the safe testing of the prototype robot cell, thereby limiting the realism of the experiments. This limitation will be addressed in future studies within a testing environment suitable for assessing the possibilities of fire and explosion hazards, and when more realistic battery modules are available for accurate testing. Also, the current iteration of the robot cell assumes no variation between products (e.g., battery module placement, component condition), which limits its robustness to uncertain variability under actual operating conditions. The authors plan to address this issue in future studies by utilising vision-based control algorithms and artificial intelligence to adapt to variability. Furthermore, the financial analysis conducted in this work is simplified due to the absence of accurate financial and operational data. A more comprehensive study, assessing the feasibility of large-scale adoption, will be undertaken in the future in collaboration with corporate partners who can supply the necessary data. Nevertheless, the proposed evaluation methodology offers a benchmark for early assessments of financial feasibility. Using this methodology, our initial assessment suggests that the robotic cell is financially viable, with a substantial risk buffer, as evidenced by the significant projected cost savings. Finally, triage of EOL batteries is also a necessary process to affect the success rate, time, and cost in the processing of EoL EV-LiBs, as module-to-cell disassembly may not always be required. However, this paper only focused on robotic disassembly technologies and assumed that triage had been done before robotic disassembly.

## Appendix

**Table 4:** A detailed overview of the proposed automated disassembly process with the required tool for each task, and considerations for generalising each step of the disassembly sequence for other battery module designs following the VDA standard

**ID**	**Task Description**	**Tool**	**Considerations for Generalisability**
**1**	**Unscrewing Task** Unscrew each screw that holds the PCB to the plastic cover. For each screw: • Move the robot such that the screwdriver tip is above the screw. • Move the screw down to engage the tool with the screw drive. • Activate the screwdriver to unfasten the screw. • Deactivate the screwdriver when the screw is fully retracted. • Place the screw into a storage box.	Screwdriver with a magnetic field.	If screws are expected for the given battery module design: • Ensure that the exact position, screw drive shape, and length for each screw are known a priori for each compatible battery module design. • Implement the unscrewing routine as a parametrised and reusable unscrewing sub-program that changes based on screw position, screw drive length, and screw length. If screws are not expected, i.e., the module has no rigid PCB: • Ensure this is known a priori and program the robot to skip the unscrewing task. Different battery module designs may feature varying screw drive types (e.g., Phillips, Torx) and sizes. • Ensure the screw drive type and size for each battery module design is known a priori, and replace the tool bit to match the screw specification for that battery module.
**2**	**Pick and place task** Pick up the circuit board and place it in a storage container. • Move the robot such that the screwdriver suction grippers are above the PCB. • Activate the suction grippers. • Move the suction gripper down until it contacts the PCB, and the suction cups have engaged with the PCB. • Move the suction gripper up to detach it from the board. • Move the suction gripper above the storage container and deactivate it to stow the PCB away.	Robotic suction gripper	If a rigid PCB is expected for the given battery module design: • Ensure the ideal positioning of the gripper on the PCB is known a priori for each compatible battery module design. • Implement the pick and place routine as a parametrised and reusable pick and place sub-program that changes based on the ideal suction gripper position. If a rigid PCB is not expected: • Ensure this is known a priori and program the robot to skip the pick-and-place task. • If a flexible PCB is present, it can be ignored as it does not need to be removed, as it does not obstruct subsequent tasks, unlike a rigid PCB.
**3**	**Cutting Task (Bus Bars)** Cut the bus bars connecting the cell terminals. Before cutting any bus bar: • Activate the grinder and move the robot to an approach position near the bus bars. For each bus bar: • Move the robot such that the grinder disk is above the bus bar. • Move the robot towards the bus bar until it touches the grinder disk. • Continue moving the grinder into the bus bar until a desired penetration depth has been reached. • Move the cutting disk along the length of the bus bar to cut it. • Move the robot such that the grinder disk is back above the bus bar. After cutting all bus bars: • Deactivate the grinder.	Grinder	Bus bars exhibit slight dimensional differences between battery module designs, resulting in variations in the start and end points of the cutting paths for each bus bar. • Ensure the differences in bus bar dimensions for the specific battery module are known a priori, and adjust the cutting path per bus bar accordingly. Depending on the number and thickness of cells, as well as the bus bar connection configuration (series and parallel), the locations and quantity of the bus bars may vary. • Ensure the number of bus bars and their locations are known a priori for each battery module design, and adjust the bus bar cutting sequence accordingly. The thickness of the bus bars may differ between battery module designs. • Ensure the bus bar thicknesses are known a priori for each battery module design, and adjust the penetration depth for the cutting operation accordingly. The bus bar cutting routine should be implemented as a parameterised and reusable cutting subprogram that changes based on the start and end points relative to the bus bar positions, the quantity and locations of the bus bars, and their thickness.
**4**	**Cutting Task (Plastic Cover)** Cut along the length of the plastic cover, near the edge of the battery module on each side, to ensure the plastic cover can be easily separated from the battery cells. Before cutting: • Activate the grinder and move the robot to an approach position above the battery module. For each side of the plastic cover: • Move the robot such that the grinder disk is above the desired side of the plastic cover. • Move the robot towards the plastic cover until it touches the grinder disk. • Continue moving the grinder into the plastic cover until a desired penetration depth has been reached. • Move the cutting disk along the length of the plastic cover to cut it. • Move the robot such that the grinder disk is back above the plastic cover. After both sides of the plastic cover: • Deactivate the grinder	Grinder	The length of the battery module can vary between different battery module designs, so the start and end points of the cutting operation will also vary. • Ensure the differences in battery module dimensions for the specific battery module are known a priori, and adjust the cutting path per battery module accordingly. The thickness of the plastic cover may differ between battery module designs. • Ensure the plastic cover thickness is known a priori for each battery module design, and adjust the penetration depth for the cutting operation accordingly. The plastic cover cutting routine should be implemented as a parameterised and reusable cutting subprogram that changes based on the module dimensions and the thickness of the plastic cover.
**5**	**Cutting Task (Casing)** Cut along the height of the module at the corner of the casing to disconnect it from the cell stack. • Move the robot such that the grinder disk is beside the corner of the casing and aligned along its height, and activate the grinder • Move the robot towards the casing until it touches the grinder disk • Continue moving the grinder into the casing until a desired penetration depth has been reached. • Move the cutting disk along the length of the casing to cut it. • Move the robot such that the grinder disk is back at the approach position, and deactivate the grinder.	Grinder	The dimensions of the battery module can vary between different battery module designs, so the start and end points of the cutting operation will also vary, as the corner location and the cutting path length will vary. • Ensure the differences in battery module dimensions for the specific battery module are known a priori, and adjust the cutting path per battery module accordingly. The thickness of the casing may differ between battery module designs. • Ensure the casing thickness is known a priori for each battery module design, and adjust the penetration depth for the cutting operation accordingly. The casing cutting routine should be implemented as a parameterised and reusable cutting subprogram that changes based on the module dimensions and the thickness of the casing.
**6**	**Bending Task** Bend open the case to extract the cells inside. • A proprietary fixture inserts pins through the casing’s through holes. • An actuator is activated that pushes the pins outwards, bending the case open.	Proprietary Bending Fixture	The dimensions of the battery module will vary, so the distances between the casing’s through holes will vary. • Design the fixture such that it can accommodate

**Table 5:** Definition of motion primitives used to construct the automated disassembly sequence in a modular way to support streamlined re-programming

** Name**	**Description**	**Operations Used In**	**Parameters**
Basic point-to-point motion	Moves the robot to the *desired joint configuration*.	Moving to approach positions before task-specific operations are executed.Ensures the robot is in the desired joint configuration before executing subsequent motions	• Desired joint configuration
Tool retrieval	Activates the tool changer to disengage the lock. Moves the robot above the *tool storage pose* and then slowly moves towards the stored tool until contact is made. Then, the tool changer is deactivated to reengage the lock. Finally, the robot moves away from the *tool storage pose* to retrieve the tool from the tool storage.	Tool changing	• Tool storage pose
Tool depositing	Moves the robot above the *tool storage pose* and then slowly moves towards the stored tool until contact is made. Then, the tool changer is activated to disengage the lock. Finally, the robot moves away from the *tool storage pose* to complete tool depositing, and then the tool changer is deactivated to reengage the lock.	Tool changing	• Tool storage pose
Unscrewing	Moves the robot-held screwdriver above the *screw pose*, moves the tool towards the screw until it is engaged, activates the screwdriver while retracting the screw, deactivates the screwdriver, and then places the screw in the assigned storage space at the defined *storage space pose*.	Unscrewing.	• Screw pose • Storage space pose
Pick and place	Moves the robot-held suction gripper above the object of interest at the *desired gripping pose*, activates the suction gripper, and moves the gripper towards the object until contact is made. The object is then lifted, and the gripper moves towards the assigned storage space at the defined *storage space pose*. Finally, the suction gripper is deactivated to deposit the object.	Pick and place for PCB extraction.	• Desired gripping position • Storage space pose
Cutting	Moves the robot-held grinder near the *cutting start pose*, activates the grinder towards the object until contact is made, and continues moving into the object until the *desired penetration depth* has been reached. Then, the grinder moves in line towards the *cutting end pose* at the desired *cutting speed*. Then, the grinder moves away from the object and is deactivated.	Cutting of the bus bars, plastic cover, and casing.	• Cutting start pose • Cutting end pose • Cutting speed • Penetration depth

**Table 6:** The speed settings used for the robot program, with descriptions of when they were used.

**Speed Setting Name**	**Robot Speed(s)**	**Description**
Fast speed	Joint speed: 100%	For motions not involving contact, such as transitioning between home and approaching certain poses.
	Linear speed: 2000 mm/s	
Mid speed	Linear speed: 500 mm/s	For non-contact motions when the robot is close to the object to be approached, this enhances safety.
Slow speed	Linear speed: 5 mm/s	For operations involving contact, such as changing tools, unscrewing, or picking up the circuit board.
Cutting speed	Linear speed: 2.5 mm/s	For cutting operations only.

## Data Availability

No datasets were generated or analysed during the current study.

## References

[1] Quebec P (2019) Lithium-ion Battery Sector: Developing a Promising Sector for Quebec’s Economy. KPMG for Propulsion Quebec. Montreal, QC, Canada

[2] Larouche F, Tedjar F, Amouzegar K, Houlachi G, Bouchard P, Demopoulos GP, Zaghib K (2020) Progress and status of hydrometallurgical and direct recycling of Li-ion batteries and beyond. Materials 13(3):801 32050558 10.3390/ma13030801 PMC7040742

[3] Skeete JP, Wells P, Dong X, Heidrich O, Harper G (2020) Beyond the event horizon: battery waste, recycling, and sustainability in the united Kingdom electric vehicle transition. Energy Res Soc Sci 69:101581

[4] Ranta V, Aarikka-Stenroos L, Mäkinen SJ (2018) Creating value in the circular economy: a structured multiple-case analysis of business models. J Clean Prod 201:988–1000

[5] Wu W et al (2020) Does energy storage provide a profitable second life for electric vehicle batteries? Energy Econ 92:105010

[6] Poschmann H, Brüggemann H, Goldmann D (2020) Disassembly 4.0: a review on using robotics in disassembly tasks as a way of automation. Chem Ing Tech 92(4):341–359. 10.1002/cite.201900107

[7] Beghi M, Braghin F, Roveda L (2023) Enhancing disassembly practices for electric vehicle battery packs: a narrative comprehensive review. Designs 7:109. 10.3390/designs7050109

[8] Harper G, Sommerville R, Kendrick E, Driscoll L, Slater P, Stolkin R, Walton A, Christensen P, Heidrich O, Lambert S, Abbott A, Ryder K, Gaines L, Anderson P (2019) Recycling lithium-ion batteries from electric vehicles. Nature 575(7781):75–86. 10.1038/s41586-019-1682-5 31695206 10.1038/s41586-019-1682-5

[9] Gerlitz E, Greifenstein M, Hofmann J, Fleischer J (2021) Analysis of the variety of lithium-ion battery modules and the challenges for an agile automated disassembly system. Procedia CIRP 96:175–180

[10] Kaarlela T, Villagrossi E, Rastegarpanah A, San-Miguel-Tello A, Pitkäaho T (2024) Robotised disassembly of electric vehicle batteries: a systematic literature review. J Manuf Syst 74:901–921

[11] Mehta A (2024) Auto sector scrambles to retool workforce for electric and automated future. Reuters. https://www.reuters.com/sustainability/climate-energy/auto-sector-scrambles-retool-workforce-electric-automated-future-2024-11-19/

[12] Krusemark L, Ganguly S, Harp T, Cullen A, Prasad S (2024) ©Center for automotive research examining workforce needs for North America: battery industry education and training needs assessment (BIETNA) 2024 pre-publication copy under review by Argonne National Laboratory and the Department of Energy. Available at: https://www.cargroup.org/wp-content/uploads/2024/02/CAR_BIETNA_2024_PreblicationCopy.pdf

[13] Kendall A, Slattery M, Dunn J (2022) Lithium-ion car battery recycling advisory group final report.vAvailable at: https://calepa.ca.gov/wp-content/uploads/2022/05/2022_AB-2832_Lithium-Ion-Car-Battery-Recycling-Advisory-Goup-Final-Report.pdf [Accessed 20 Jun 2025]

[14] Larouche F, Demopoulos GP, Amouzegar K, Bouchard P, Zaghib K (2018) Recycling of Li-ion and Li-solid state batteries: the role of hydrometallurgy. InExtraction. : Proceedings of the First Global Conference on Extractive Metallurgy 2018 (pp. 2541–2553). Springer International Publishing

[15] Friedrich B, Peters L Status and Trends of industrialised Li-Ion battery recycling processes with qualitative comparison of economic and environmental impacts. InProceedings of the 22nd International Congress for Battery Recycling ICBR, Lisbon, Portugal 2017 Sep (pp. 20–22)

[16] Thompson D, Hyde C, Hartley JM, Abbott AP, Anderson PA, Harper GDJ (2021) To shred or not to shred: a comparative techno-economic assessment of lithium ion battery hydrometallurgical recycling retaining value and improving circularity in LIB supply chains. Resour Conserv Recycl 175:105741. 10.1016/j.resconrec.2021.105741

[17] Harper G, Sommerville R, Kendrick E, Driscoll L, Slater P, Stolkin R, Anderson P (2019) Recycling lithium-ion batteries from electric vehicles. Nature 575(7781):75–86 31695206 10.1038/s41586-019-1682-5

[18] Meng K, Xu G, Peng X, Youcef-Toumi K, Li J (2022) Intelligent disassembly of electric-vehicle batteries: a forward-looking overview. Resour Conserv Recycl 182:106207. 10.1016/j.resconrec.2022.106207

[19] Wu T, Zhang Z, Yin T, Zhang Y (2022) Multi-objective optimisation for cell-level disassembly of waste power battery modules in human-machine hybrid mode. Waste Manag 144:513–526. 10.1016/j.wasman.2022.04.015 35468449 10.1016/j.wasman.2022.04.015

[20] Yu J, Zhang H, Jiang Z, Yan W, Wang Y, Zhou Q (2022) Disassembly task planning for end-of-life automotive traction batteries based on ontology and partial destructive rules. J Manuf Syst 62:347–366. 10.1016/j.jmsy.2021.12.006. /01/01/, doi

[21] Alfaro-Algaba M, Ramirez FJ (2020) Techno-economic and environmental disassembly planning of lithium-ion electric vehicle battery packs for remanufacturing. Resour Conserv Recycling 154:104461. 10.1016/j.resconrec.2019.104461

[22] Wu T, Zhang Z, Zeng Y, Zhang Y, Guo L, Liu J (2024) Techno-economic and environmental benefits-oriented human–robot collaborative disassembly line balancing optimisation in remanufacturing. Robotics and Computer-Integrated Manufacturing 86:102650. 10.1016/j.rcim.2023.102650

[23] Laili Y, Wang Y, Fang Y, Pham DT (2022) Optimisation of robotic disassembly for remanufacturing. Springer

[24] Lee M-L, Liu W, Behdad S, Liang X, Zheng M (2022) Robot-assisted disassembly sequence planning with real-time human motion prediction. IEEE Trans Syst Man Cybernetics: Syst 53(1):438–450

[25] Zhang Y, Lu H, Pham DT, Wang Y, Qu M, Lim J, Su S (2019) Peg–hole disassembly using active compliance. R Soc Open Sci 6(8):190476. 10.1098/rsos.190476 31598244 10.1098/rsos.190476 PMC6731726

[26] Qu M, Wang Y, Pham DT (2023) Robotic disassembly task training and skill transfer using reinforcement learning. IEEE Trans Ind Inform. 10.1109/TII.2023.3242831

[27] Li R, Pham DT, Huang J, Tan Y, Qu M, Wang Y, Kerin M, Jiang K, Su S, Ji C Unfastening of hexagonal headed screws by a c

[28] Chen W, Hua K, Wegener, Dietrich F (2014) A robot assistant for unscrewing in hybrid human-robot disassembly. (2014) IEEE International Conference on Robotics and Biomimetics (ROBIO 2014). IEEE

[29] Li R et al (2020) Unfastening of hexagonal headed screws by a collaborative robot. IEEE Trans Autom Sci Eng 17(3):1455–1468

[30] Huang J et al (2020) Strategies for dealing with problems in robotised unscrewing operations. International precision assembly seminar. Springer International Publishing, Cham

[31] Elguea-Aguinaco Íñigo et al (2022) Goal-conditioned reinforcement learning within a human-robot disassembly environment. Appl Sci 12:11610

[32] Qu M, Wang Y, Pham Duc Truong (2023) Robotic disassembly task training and skill transfer using reinforcement learning. IEEE Trans Ind Inform 19:10934–10943

[33] Vongbunyong S, Chen WH (2015) Automated systems with cognitive abilities. Springer, Cham,, Disassembly Automation

[34] Chen WH, Foo G, Kara S, Pagnucco M (2021) Automated generation and execution of disassembly actions. Robotics and Computer-Integrated Manufacturing 68:102056. 10.1016/j.rcim.2020.102056

[35] Huang J, Pham DT, Li R, Qu M, Wang Y, Kerin M, Su S, Ji C, Mahomed O, Khalil R (2021) An experimental human-robot collaborative disassembly cell. Comput Ind Eng 155:107189

[36] Huang J, Pham DT, Wang Y, Qu M, Ji C, Su S, Xu W, Liu Q, Zhou Z (2020) A case study in human– robot collaboration in the disassembly of press-fitted components. Proceedings of the Institution of Mechanical Engineers, Part B: Journal of Engineering Manufacture 234(3):654–664

[37] Wegener K, Chen WH, Dietrich F, Dröder K, Kara S (2015) Robot assisted disassembly for the recycling of electric vehicle batteries. Procedia CIRP, vol 29 pp 716–721, 2015/01/01/. 10.1016/j.procir.2015.02.051

[38] Wegener K, Andrew S, Raatz A, Dröder K, Herrmann C (2014) Disassembly of electric vehicle batteries using the example of the Audi Q5 hybrid system. Procedia CIRP 23:155–160. 10.1016/j.procir.2014.10.098

[39] Tan WJ, Chin CMM, Garg A, Gao L (2021) A hybrid disassembly framework for disassembly of electric vehicle batteries. Int J Energy Res 45(5):8073–8082

[40] Fleischer J, Gerlitz E, Rieβ S, Coutandin S, Hofmann J (2021) Concepts and requirements for flexible disassembly systems for drive train components of electric vehicles. Procedia CIRP 98:577–582. 10.1016/j.procir.2021.01.154

[41] Poschmann H, Brüggemann H, Goldmann D (2021) Fostering end-of-life utilization by information driven robotic disassembly. Procedia CIRP 98:282–287. 10.1016/j.procir.2021.01.104

[42] Qu M, Pham DT, Altumi F, Gbadebo A, Hartono N, Jiang K, Kerin M, Lan F, Micheli M, Xu S, Wang Y (2024) Robotic disassembly platform for disassembly of a plug-in hybrid electric vehicle battery: a case study. Automation 5(2):50–67

[43] Choux M, Pripp SW, Kvalnes F, Hellström M (2024) To shred or to disassemble–a techno-economic assessment of automated disassembly vs. shredding in lithium-ion battery module recycling. Resour Conserv Recycl 203:107430

[44] Villagrossi E, Dinon T (2023) Robotics for electric vehicles battery packs disassembly towards sustainable remanufacturing. Jnl Remanufactur 13:355–379. 10.1007/s13243-023-00134-z

[45] Els F (2025) CHART: Prismatic cells capture 69% of global EV battery market - Adamas Intelligence. Adamas Intelligence. https://www.adamasintel.com/chart-prismatic-cells-capture-69-of-global-ev-battery-market/

[46] 48v Audi Q5 PHEV Modules, Cells S (2021) JAG35, jag35.com/products/48v-audi-q5-phev-modules-zero-miles-samsung-cells. Accessed 21 June 2025

[47] ContentKeeper Content Filtering. Evshop.eu (2025) evshop. http://eu/en/batteries/1074-12s-25ah-vw-gte-11kwh-48v-battery-module.html. Accessed 21 June 2025

[48] Ford Kuga III (2023) Battery cell module for hybrid/electric cars 112KW AMD193834. EBay UK, 2023. www.ebay.co.uk/itm/306331960945. Accessed 21 June 2025

[49] Hybrid Battery Cell Pack LEV40 LEV50 for Mitsubishi Outlander Mk3 PHEV (2025). EBay UK. www.ebay.co.uk/itm/306339255272. (**Accessed 21 June 2025.**)

[50] Mg Maxus Hybrid CATL Battery Li- (2024) Ion 60v 2.77kWH solar power wall motor home. EBay UK. www.ebay.co.uk/itm/226379174818. (**Accessed 21 June 2025.**)

[51] *News Release*. (2024) www.blue-energy.co.jp/en/newsrelease/. Accessed 21 June 2025

[52] Peugeot E (2024) Boxer NMC Cells Reuse Modules. Openinverter Forum. http://openinverter.org/forum/viewtopic.php?t = 4801. Accessed 21 June 2025

[53] Porsche Panamera 971 Cayenne Audi vw Battery Module. www.xdalys.lt/en/product/14160308309/

[54] Samsung Battery 12S1P 43.2V 50ah electric car battery for nissan leaf battery modules solar system EV RV (2021). www.alibaba.com/product-detail/Samsung-battery-12S1P-43-2V-50ah_1600537279361.html. Accessed 21 June 2025

[55] The Power behind MG’s Battery Technology (2020) MG Motor Europe, 8 Dec. news.mgmotor.eu/the-power-behind-mg-battery-technology/

[56] Battery VWE-G (2025) Module 1.63kWh 14.8V (48v Use). Zoomev, zoomev. http://uk/products/vw-e-golf-battery-module-1-63kwh-14-8v-4s-111ah-48v. Accessed 21 June 2025

[57] Blue Energy Co (2024) www.blue-energy.co.jp/en/. Accessed 21 June 2025

[58] Shop EV evshop. http://eu/en/batteries/153-53kwh-bmw-i3-battery-module-45v-120ah-nominal.html. Accessed 21 June 2025

[59] CALB 3P4S lithium NMC EV battery modules shipped to the UK. Evlithium (2025) www.evlithium.com/order-and-shipment/calb-ev-battery-module-to-the-uk.html. Accessed 21 June 2025

[60] China CATL 12s1p Ev Rv 44.4v 37Ah lithium ion electric car 50ah rechargeable Nmc Li Ion battery module for electric car battery manufacturer and supplier | UPOWER (2020). www.uli-power.com/catl-12s1p-ev-rv-44-4v-37ah-lithium-ion-electric-car-50ah-rechargeable-nmc-li-ion-battery-module-for-electric-car-battery-product/. Accessed 21 June 2025

[61] Hotsale top brand new 12s1p 50ah nmc battery module vda 355 car batteries for leaf battery car batte (2025). NOGI Battery. nogibattery.com/products/wholesale-12s1p-432v-50ah-electric-car-battery-for-leaf-battery-modules-solar-system-ev-rv. Accessed 21 June 2025

[62] Shop EV evshop.eu/en/battery-module/1744-6s2p-stellantis-battery-module.html . Accessed 21 June 2025.

[63] Towards independent innovation: power battery from vda, meb to short knife | EVlithiumcharger (2018). www.evlithiumcharger.com/News/power-battery-from-vda-to-short-knife.html. Accessed 21 June 2025

[64] What is VDA355 and MEB590 module༟ (2025) Lythbattery Com. lythbattery.com/what-is-vda355-and-meb590-module. Accessed 21 June 2025.

[65] Battery module NOGI battery (2025). nogibattery.com/collections/battery-module. Accessed 21 June 2025.

[66] Desai A, Mital A (2003) Evaluation of disassemblability to enable design for disassembly in mass production. Int J Ind Ergon 32(4):265–281. 10.1016/S0169-8141(03)00067-2

[67] Tan WJ, Chin CMM, Garg A, Gao L (2021) A hybrid disassembly framework for disassembly of electric vehicle batteries. Int J Energy Res 45(5):8073–8082. 10.1002/ER.6364

[68] Kroll E, Hanft TA (1998) Quantitative evaluation of product disassembly for recycling. Res Eng Des 10(1):1–14. 10.1007/BF01580266/METRICS

[69] Peeters JR, Vanegas P, Mouton C, Dewulf W, Duflou JR (2016) Tool design for electronic product dismantling. Procedia CIRP 48:466–471. 10.1016/J.PROCIR.2016.03.106

[70] Average salary checker (no date) Jobs Available at: https://www.reed.co.uk/average-salary/average-engineer-technician-salary (Accessed: 10 September 2024)

[71] University of Birmingham post-doctoral fellow salaries (No date) Available at: https://uk.indeed.com/cmp/University-of-Birmingham/salaries/Post-doctoral-Fellow (Accessed: 10 September 2024)

[72] Johns K (2024) 10 financial metrics every A&E firm should track, Wipfli. https://claytonmckervey (Accessed: 10 September 2024)

[73] Energy price cap (no date) Ofgem Available at: https://www.ofgem.gov.uk/energy-price-cap (Accessed: 10 September 2024)

[74] Lefley F (1996) The payback method of investment appraisal: a review and synthesis. Int J Prod Econ 44(3):207–224

[75] ATARI Research Team. Demonstration of robotic disassembly of an EV battery module [Internet]. The University of Birmingham (2023) Oct 30 [cited 2025 Feb 9]. Available from: https://www.youtube.com/watch?v=A12RFlvI0Lk

